# Solvent Extraction and Identification of Active Anticariogenic Metabolites in *Piper cubeba* L. through ^1^H-NMR-Based Metabolomics Approach

**DOI:** 10.3390/molecules23071730

**Published:** 2018-07-16

**Authors:** Raja Nur Asila Raja Mazlan, Yaya Rukayadi, M. Maulidiani, Intan Safinar Ismail

**Affiliations:** 1 Laboratory of Natural Products, Institute of Bioscience, Universiti Putra Malaysia, Serdang 43400, Malaysia; asila.mazlan@gmail.com (R.N.A.R.M.); maulidiani@upm.edu.my (M.M.); safinar@upm.edu.my (I.S.I.); 2 Department of Food Science, Faculty of Food Science and Technology, Universiti Putra Malaysia, Serdang 43400, Malaysia; 3 Department of Chemistry, Faculty of Science, Universiti Putra Malaysia, Serdang 43400, Malaysia

**Keywords:** anticariogenic, anti-inflammatory, dental caries, multivariate data analysis, ^1^H-Nuclear Magnetic Resonance (NMR)

## Abstract

The aim of this study was to determine the effects of different solvents for extraction, liquid–liquid partition, and concentrations of extracts and fractions of *Piper cubeba* L. on anticariogenic; antibacterial and anti-inflammatory activity against oral bacteria. Furthermore, ^1^H-Nuclear Magnetic Resonance (NMR) coupled with multivariate data analysis (MVDA) was applied to discriminate between the extracts and fractions and examine the metabolites that correlate to the bioactivities. All tested bacteria were susceptible to *Piper cubeba* L. extracts and fractions. Different solvents extraction, liquid–liquid partition and concentrations of extracts and fractions have partially influenced the antibacterial activity. MTT assay showed that *P. cubeba* L. extracts and fractions were not toxic to RAW 264.7 cells at selected concentrations. Anti-inflammatory activity evaluated by nitric oxide (NO) production in lipopolysaccharide (LPS) stimulated cells showed a reduction in NO production in cells treated with *P. cubeba* L. extracts and fractions, compared to those without treatment. Twelve putative metabolites have been identified, which are (**1**) cubebin, (**2**) yatein, (**3**) hinokinin, (**4**) dihydrocubebin, (**5**) dihydroclusin, (**6**) cubebinin, (**7**) magnosalin, (**8**) *p*-cymene, (**9**) piperidine, (**10**) cubebol, (**11**) d-germacrene and (**12**) ledol. Different extraction and liquid–liquid partition solvents caused separation in principal component analysis (PCA) models. The partial least squares (PLS) models showed that higher anticariogenic activity was related more to the polar solvents, despite some of the active metabolites also present in the non-polar solvents. Hence, *P. cubeba* L. extracts and fractions exhibited antibacterial and anti-inflammatory activity and have potential to be developed as the anticariogenic agent.

## 1. Introduction

Dental caries is a noticeable infection in humans and becomes a major inconvenience for health provider companies [1]. Dental caries occurs when pathogenic bacteria produce an acid through a fermentation process from food particles aggregated on the tooth surface which promotes tooth demineralization [2]. People are vulnerable to this disease at some stage in their lifetime. This disease affects children and adults on the primary and permanent teeth at coronal and root surfaces. A current overview of the accessible epidemiological records from numerous nations demonstrates that there is an increment of dental caries cases [3].

Considerable efforts have been made to eliminate dental caries, for example, elimination of the dental plaque by mechanical method [4], and dental treatment and through utilizing chemical compounds such as chlorhexidine. However, these approaches do not successfully reduce the occurrence of dental caries because of unaffordability to the high cost incurred for dental treatment and less accessibility for people in rural and remote areas [5]. Furthermore, frequent usage of dental products consisting of chemical compounds is regularly related to several side effects such as tooth and restoration staining, modification in the flavor of foods, and a burning sensation at the tip of the tongue [5,6,7]. With these issues arose, therefore, the importance of efforts to discover new and safe anticariogenic agents.

The bacteria associated with dental caries are often referred to as cariogenic bacteria. If left untreated, dental caries can give rise to pulpitis, a disease related to an inflammation of the dental pulp. Therefore, to prevent dental caries and pulpitis, the study of plants’ derivatives with anticariogenic properties which include both antibacterial and anti-inflammatory activities are required. For many years, *P. cubeba* L. dried berries have been used in food preparations as well as for medicinal purposes. An approach of using different solvents for extraction, liquid–liquid partition, and different doses of extracts and fractions might be useful in understanding the antibacterial activity at its best. Hence, the antibacterial activity of *P. cubeba* L. dried berries extracts and fractions in different solvents and concentrations were tested against oral bacteria in disc diffusion (DDA), minimum inhibitory concentration (MIC) and minimum bactericidal concentration (MBC) assays. For anti-inflammatory activity, nitric oxide (NO) production in lipopolysaccharide (LPS) stimulated RAW 264.7 was carried out on the *P. cubeba* L. dried berries samples.

Even though the usage of natural products has been widely accepted by the community, there is still concern regarding their safety. Toxicity of the extracts and fractions of *P. cubeba* L. needs to be determined particularly for safe usage in oral administration. Thus, 3-(4,5-dimethylthiazol-2-yl)-2,5-diphenyltetrazolium bromide (MTT) cells viability assay was undertaken. As metabolomics permits a holistic approach to investigate an intricate mixture of phytochemicals which can be linked to observations acquired via biological testing systems without the necessity of isolation work [8], herein, the characterization of active anticariogenic metabolites through proton Nuclear Magnetic Resonance (^1^H-NMR) coupled with multivariate data analysis (MVDA) is described.

## 2. Results

### 2.1. Yield of P. cubeba *L*. Extracts

The extraction of dried berries of *P. cubeba* L. was carried out in three different solvents of methanol, ethanol and hexane which yielded crude extracts of dark brown and viscous liquid. The extraction yields are tabulated in Table 1.

### 2.2. Antibacterial Activity of P. cubeba *L*. Extracts

#### 2.2.1. Disc Diffusion Assay 

The principle of disc diffusion assay (DDA) is the larger the inhibition zone, the greater the antibacterial activity. In this study, DDA was performed to screen the antibacterial activity of *P. cubeba* L. extracts in range of concentrations (6.3 to 100 mg/mL) against three oral strains of Gram-positive bacteria, which are *Streptococcus mutans* KCCM3309, *S. sobrinus* ATCC33478 and *Actinomyces viscosus* ATCC15987. DDA results of the *P. cubeba* L. extracts are presented in Table 2.

In all extracts each of five different concentrations were used to determine their activity against three strains of oral bacteria. The bacteria exhibited different susceptibility against different *P. cubeba* L. extracts. The highest activity recorded was in ethanol extract with inhibition value 10.33 mm towards *S. mutans*, while there was 12.17 mm of inhibition value for methanol extract towards *S. sobrinus* and 12.67 mm for hexane extract towards *A. viscosus*. For the test against *S. mutans*, different concentrations of methanol and ethanol extracts have not significantly contributed to differences in inhibition values. However, inhibition zones were significantly different between the lowest hexane extract concentration; 6.3 mg/mL with the rest of the hexane extract concentrations (12.5, 25.0, 50, and 100 mg/mL). The inhibition zone values for *S. mutans* between different solvents were not significantly different at the highest concentration of 100 mg/mL. For the test against *S. sobrinus*, different concentrations of methanol, ethanol and hexane extracts had significantly contributed to the differences in inhibition zone values. At the highest concentration of the extracts; 100 mg/mL, the inhibition zone values of methanol and hexane extracts were significantly different from those of ethanol extracts. Meanwhile, for the test against *A. viscosus*, different concentrations even at the highest dose of all the extracts; methanol, ethanol and hexane did not significantly affect the inhibition zone values. In summary, different extracts at a range of concentrations (6.3 mg/mL to 100.0 mg/mL) did not influence the inhibition zone values in the DDA test against *S. mutans* and *A. viscosus*. Only hexane extract showed a significant inhibition zone value when tested against *S. mutans*. At the highest extract concentration, different types of solvent extraction also did not influence the inhibition zone values of *S. mutans* and *A. viscosus*. On the other hand, the inhibition zone values observed for the test against *S. sobrinus* showed a significant difference in activity, when tested using different extract concentrations. As for the test against *S. sobrinus,* at the highest extract concentrations, different types of solvent extraction did influence the inhibition zone values. The data obtained from the DDA, however, is inadequate as the assay provides only qualitative data and acts as a preliminary screening. Moreover, the hydrophobic nature of most essential oils (EOs) and plant extracts could prevent the uniform diffusion of these substances through agar medium [9,10] whereby the antibacterial activity of the extracts could not be accurately measured through this assay. Therefore, MIC and MBC determination are necessary to further conducted to confirm the antibacterial activity of the *P. cubeba* L. extracts.

#### 2.2.2. Minimum Inhibitory Concentration (MIC) and Minimum Bactericidal Concentration (MBC)

MIC is defined as the minimum concentration needed to inhibit at least 99% of bacteria growth (bacteriostatic). MBC is defined as the minimum concentration of the plant extracts required to kill at least 99% of the bacteria (bactericidal) [11]. MIC and MBC values of three *P. cubeba* L. extracts against three strains of oral bacteria are summarized in Table 3.

*Piper cubeba* L. extracts exerted greater bactericidal activity towards *S. mutans*, as compared to *S. sobrinus* and *A. viscosus*. *Streptococcus sobrinus* seems to be more sucrose-dependent than *S. mutans* and is capable of producing more acids, making it more difficult to kill [12,13,14]. The results from a previous study also highlighted that *A. viscosus* took longer to kill compared to *S. mutans* [15]. Ethanol and hexane extracts showed good bactericidal activity towards *S. mutans*, while methanol extract exhibited higher activity towards *S. sobrinus*. On the other hand, *A. viscosus* was more susceptible to hexane extract. As previously reported, *P. cubeba* L. was known to produce various types of phenolic lignans [16], and thus it can be postulated that the antibacterial activity of the methanol and ethanol extracts was probably owing to these phenolic compounds. It is noteworthy that *P. cubeba* L. also produces mono- and sesquiterpenes, which are mostly non-polar or medium polarity in nature. These terpenoids are likely to be present in the hexane extract which probably could contribute to the antibacterial activity [16]. However, the MIC and MBC of the positive control (CHX) showed lower values in comparison to those of *P. cubeba* L. extracts.

### 2.3. Liquid–Liquid Partition of P. cubeba *L*. Extract

Methanol crude extract of *P. cubeba* L. was selected to be further fractionated by liquid–liquid partition, since this extract gave a consistent yield and suitable for extracting polyphenolic compound [17]. Table 4 shows yields from the liquid–liquid partition of *P. cubeba* L. methanol extract.

### 2.4. Antibacterial Activity of P. cubeba *L*. Fractions

#### 2.4.1. Disc Diffusion Assay

Disc diffusion assay (DDA) of fractions against oral bacteria data are summarized in Table 5.

From Table 5, the highest activity was recorded in aqueous methanol fraction with inhibition zone values recorded at 11.00, 11.20 and 12.33 mm, when tested against *S. mutans*, *S. sobrinus* and *A. viscosus*, respectively. For the test against *S. mutans*, inhibition zone values of hexane and ethyl acetate fractions showed differences between the lowest concentration; 6.3 mg/mL with the rest of the extract concentrations (12.5, 25.0, 50.0 and 100 mg/mL). Inhibition zone values were also different when tested using different concentrations of aqueous methanol fraction. At the highest concentration of the fractions; 100 mg/mL, the inhibition zone value of an aqueous methanol fraction was notably different from ethyl acetate and hexane fraction. Different concentrations of hexane, ethyl acetate and aqueous methanol fractions contributed to the remarkably difference in inhibition zone values for the test against *S. sobrinus*. However, at the highest concentration (100 mg/mL) of the fractions, the inhibition zone values were not significantly different. For the test against *A. viscosus*, different concentrations of all the fractions; hexane, ethyl acetate and aqueous methanol notably affected the inhibition zone values. At the highest concentration of 100 mg/mL for all fractions, the inhibition zone values’ difference of hexane and aqueous methanol fractions from ethyl acetate fraction were apparent. In summary, inhibition zone values of *S. mutans*, *S. sobrinus* and *A. viscosus* were influenced by different fractions’ concentrations. At the highest concentration, even different types of solvent fractions showed influence on the inhibition zone values against *S. mutans* and *A. viscosus*, except for *S. sobrinus*. MIC and MBC determination will be further evaluated on the antibacterial activity of the *P. cubeba* L. fractions.

#### 2.4.2. Minimum Inhibitory Concentration (MIC) and Minimum Bactericidal Concentration (MBC)

MIC and MBC values of three *P. cubeba* L. fractions against three strains of oral bacteria are summarized in Table 6.

*Piper cubeba* L. fractions exhibited the best bactericidal activity towards *S. mutans* as compared to *S. sobrinus* and *A. viscosus*. Hexane and aqueous methanol fractions demonstrated greater bactericidal activity towards *S. mutans,* and only aqueous methanol fraction has notable activity against *S. sobrinus* and *A. viscosus*. Antimicrobial properties of *P. cubeba* L. extracts and fractions were attributed by the compounds that are present in the berries. Dried berries of *P. cubeba* L. have a high composition of alkaloids, lignans and essential oils (EOs) [18,19]. Interestingly, alkaloids possess the ability to link with bacterial DNA, and then kill the bacteria [20]. On the other hand, phenolic compounds were found to have precipitative activity on microbial enzymes which leads to the microbial inhibition and loss of function [21]. Cowan [22] reported that EOs exhibited antimicrobial activity through the disruption of the bacteria cell membrane’s function. The significant activity contributed by hexane fraction towards *S. mutans* might be attributed to the non-polar compounds present in *P. cubeba* L. [16]. However, MIC and MBC of *P. cubeba* L. fractions is not comparable to that of the positive control (CHX) which showed lower values for both test. 

### 2.5. Nitric Oxide (NO) Production by Extracts and Fractions of P. cubeba *L*. in Lipopolysaccharide (LPS) Stimulated RAW 264.7 Cells

**Figure 1:**
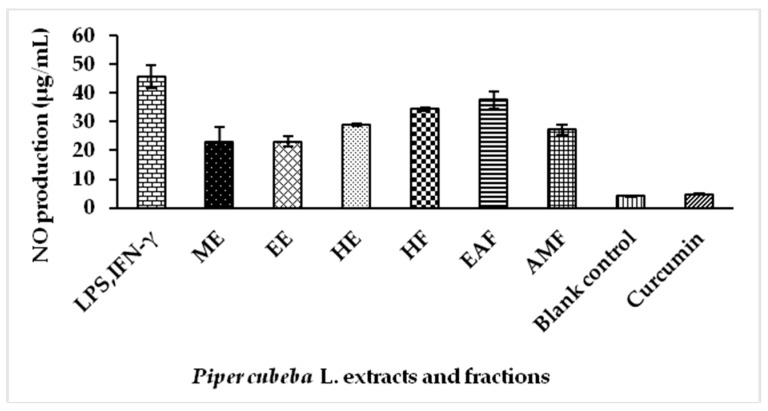
NO production by *P. cubeba* L. extracts and fractions of in lipopolysaccharide (LPS) stimulated RAW 264.7 cells at 62.5 µg/mL.ME: methanol extract; EE: ethanol extract; HE: hexane extract; HF: hexane fraction; EAF: ethyl acetate fraction; AMF: aqueous methanol fraction.

Inflammation is a mechanism of innate immunity, which is defined as a biological response process of vascular tissues to harmful stimuli, for example, damaged cells, irritants and pathogens [23]. Nitric oxide (NO) is an inorganic free radical that is involved in physiological and pathological processes and becomes one of the inflammatory mediators that triggers inflammation in many organs. At lower concentrations, NO has been demonstrated to play a role as a neurotransmitter. However, NO produced at higher concentrations is implicated to have a role in acute or chronic inflammation, ischemic stroke, non-specific host defense, pathogenesis of vasodilation and septic shock [24,25]. In the present study, the NO production by extracts and fractions of *P. cubeba* L. stimulated with LPS and IFN-γ in RAW 264.7 cells were evaluated. The data are summarized in Figure 1.

The NO production in the cells increased drastically, when stimulated by LPS and IFN-γ. At the concentration of 62.5 µg/mL, pre-treated cells showed lower NO production, as compared to the untreated supernatants (Figure 1). Methanol extract produced the lowest NO, which was 22.98 µg/mL followed by ethanol extract, 23.13 µg/mL and aqueous methanol fraction, 27.31 µg/mL. NO production for LPS-stimulated cells, without treatment, was recorded at 45.53 µg/mL. NO production of positive control, curcumin at concentration (2 µL/mL) was recorded at 4.79 µg/mL (Supplementary Table S2). *Piper cubeba* L. extracted or partitioned with polar solvents showed substantial reduction of NO compared to the non-polar solvents. The result of MTT cell viability assays revealed that the inhibitory effect of methanol extract was not due to cell damage (viability > 80%) (Supplementary Figures S1 and S2). Phenolic compounds (present in polar extraction/partition solvents) are known to be potent for inhibiting NO and peroxynitrite productions [26]. This result may be significantly explained by the possible interaction between phenolic compounds and LPS. It has been postulated that the phenolic compounds might bind to the LPS and prevent it from successfully initiating inflammation in the cells [27,28,29,30]. 

### 2.6. Putative Identification of Metabolites in Extracts and Fractions of P. cubeba *L*. Based on ^1^H-Nuclear Magnetic Resonance (NMR)

**Figure 2:**
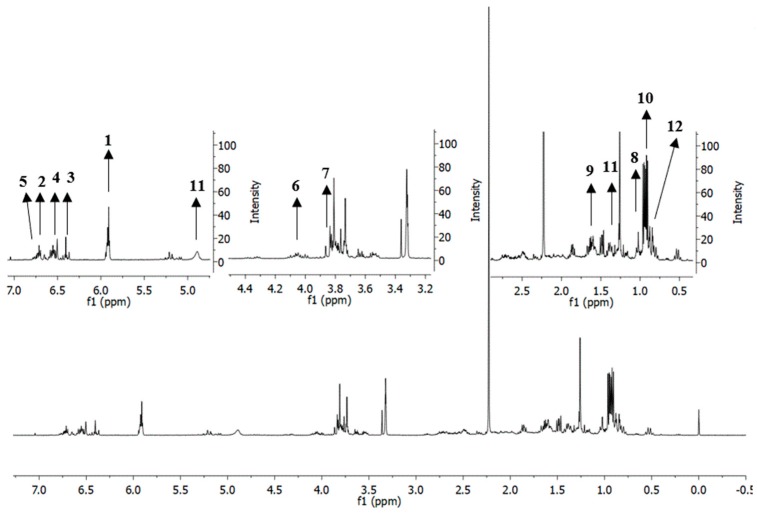
The representative ^1^H-NMR spectrum of methanol extract of *P. cubeba* L. The numbers indicate the identified putative metabolites. **1**: cubebin; **2**: yatein; **3**: hinokinin; **4**: dihydrocubebin; **5**: dihydroclusin; **6**: cubebinin; **7**: magnosalin; **8**: *p*-cymene; **9**: piperidine; **10**: cubebol; **11**: d-germacrene; **12**: ledol.

Metabolites in the different solvents extractions and liquid–liquid partitions were putatively identified by using ^1^H-NMR data obtained by using 500 MHz Varian INOVA NMR spectrometer. Figure 2 illustrates the ^1^H-NMR representative spectrum of *P. cubeba* L. methanol extract.

From the visual inspection of the spectra, there was no significant difference in the number of identified metabolites between all the extracts and fractions of *P. cubeba* L (Supplementary Figures S3–S7). However, the samples differed in the intensity level of the signals produced. Extracts of methanol, ethanol, hexane and hexane fraction showed higher intensity of signals in the upfield region of the spectra between δ 0.5 to δ 2.0 ppm which corresponded to an aliphatic region [31]. These signals could be related to EOs group in which *p*-cymene, cubebol, d-germacrene and ledol are among EOs produced by *P. cubeba* L. Meanwhile, ethyl acetate fraction, followed by aqueous methanol fraction, methanol extract and ethanol extracts exhibited highest overlapping signals in the downfield region or aromatic region [31] between δ 6.40 to δ 6.80 ppm. It is suggested that these signals correlate with the lignans group such as cubebin, yatein, hinokinin, dihydrocubebin, dihydroclusin, cubebinin and magnosalin. All the identified putative metabolites are listed in Table S1 (Supplementary Materials). In summary, 12 putative metabolites in all *P. cubeba* L. samples have been successfully identified as (**1**) cubebin, (**2**) yatein, (**3**) hinokinin, (**4**) dihydrocubebin, (**5**) dihydroclusin, (**6**) cubebinin, (**7**) magnosalin, (**8**) *p*-cymene, (**9**) piperidine, (**10**) cubebol, (**11**) d-germacrene and (**12**) ledol. These metabolites are known phytoconstituents of *P. cubeba* based on the previously reported studies [32,33,34,35,36]. The identification of these metabolites was performed by comparison of their ^1^H-NMR spectral data with those in the literature review. Further confirmation by 2-dimensional (2D) ^1^H *J*-resolved (JRES) NMR spectroscopy could not be fully achieved as there were certain signals which did not clearly appeared in 2D JRES NMR spectra. Nevertheless, the identity of the metabolites was confirmed by 2D heteronuclear multiple bond correlation (HMBC) NMR spectroscopy (Supplementary Figure S8a,b). There was no significant difference in the number of identified metabolites when using different solvents (methanol, ethanol and hexane) for extraction; and liquid–liquid partition (hexane, ethyl acetate and aqueous methanol). However, there were clear differences in the signals intensity level produced by these samples.

### 2.7. Multivariate Data Analysis (MVDA)

The measurement of metabolites within an organism using quantitative and qualitative methods subjected to certain factors is described as metabolomics [37]. Nowadays, many studies prefer to use NMR as it allows immediate identification of the different groups of secondary and primary metabolites. Moreover, this method is reproducible, rapid and involves only minimal sample preparation. Currently, many studies have coupled the NMR techniques with the MVDA for metabolic profiling and characterization purposes [38,39]. In this study, *P. cubeba* L. extracts and fractions were subjected to different solvents for extraction and liquid–liquid partition and the metabolites’ variation were observed using the ^1^H-NMR dataset coupled with MVDA. The correlation between antibacterial activity and NO anti-inflammatory activity to the metabolites of *P. cubeba* L. was also determined.

#### 2.7.1. Principal Component Analysis (PCA)

Principal component analyses (PCA) were performed in order to conduct an investigation in a bias-free manner the suitability of the present data set for MVDA. As expected, the extracts and fractions obtained from solvents of different polarity formed clusters in the scores plots and some of the identified constituents having different polarity could be associated with the major differences between the samples (Supplementary Figures S9 and S10a,b). No significant outlier was observed. Spectral relative standard deviation (RSD) values for extracts were summarized in Supplementary Figures S11 and S12. The RSD results showed the acceptable values ranged from 46.82% to 75.88% for all extracts.

#### 2.7.2. Partial Least Squares (PLS)

Partial least squares (PLS), also known as projection to latent structure (PLS), a supervised MVDA, was applied to understand the relationship between biological activities and the metabolites of different extracts and fractions of *P. cubeba* L. Biplotting involved both score and loading plots, in which the sample differences and the variable accountable for the separation can be deduced from this plot [40,41]. In this study, the biplot PLS model explained the correlation among the X or independent variables of metabolites NMR chemical shifts, to the Y or dependent variables which were antibacterial activity (1/MBC) toward *S. sobrinus* and the NO anti-inflammatory activity assay (1/NO).

**Figure 3:**
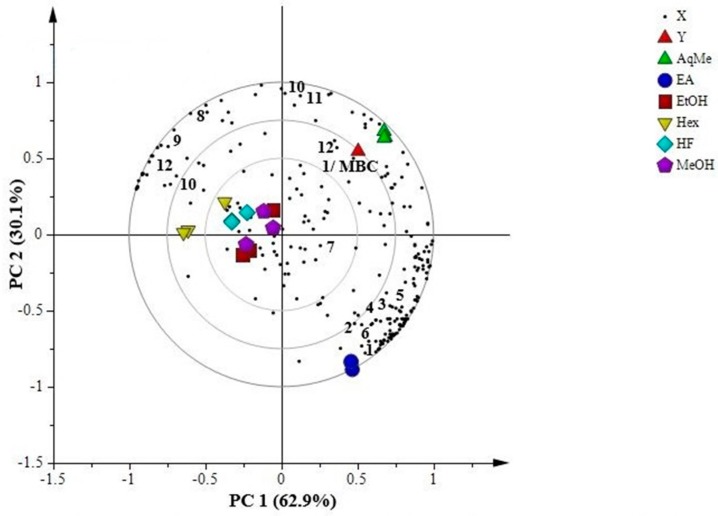
The biplot demonstrates the correlation between extracts and fractions of *P. cubeba* L. with antibacterial activity of minimum bactericidal concentration (MBC) towards *S. sobrinus*. MeOH: methanol extract; EtOH: ethanol extract; Hex: hexane extract; HF: hexane fraction; EA: ethyl acetate fraction; AqMe: aqueous methanol fraction. The numbers indicate the identified putative metabolites. **1**: cubebin; **2**: yatein; **3**: hinokinin; **4**: dihydrocubebin; **5**: dihydroclusin; **6**: cubebinin; **7**: magnosalin; **8**: *p*-cymene; **9**: piperidine; **10**: cubebol; **11**: d-germacrene; **12**: ledol. X: metabolites in *P. cubeba* L., Y: 1/MBC.

Figure 3 shows the PLS biplot that represents correlation between antibacterial activity (MBC) with the respective NMR data. The aqueous methanol fraction located close to the 1/MBC position on the right upper quadrant, suggesting high correlation between the fraction and antibacterial activity. There were only three identified metabolites in the same quadrant as the aqueous methanol fraction, which were cubebol, d-germacrene and ledol. Other metabolites in the same quadrant are still unidentified but believed to also be responsible for the antibacterial activity exhibited by the aqueous methanol fraction. The identified metabolites were associated with the EOs. The EOs were typically liquid, clear and unusually coloured, complex and the present compounds are volatile, characterized by a strong odour. This group of metabolites are synthesized by aromatic plants into secondary metabolites, which acts to protect the plant against microorganisms and insects [42,43]. Most EOs are composed of terpenes, terpenoids, and other aromatic and aliphatic constituents with low molecular weights [44,45]. Various mechanisms of antibacterial activity of EOs have been proposed, including the disruption of the cellular membrane which has effects on both the external envelope of the cell and the cytoplasm, and reduces the membrane potential [44]. Several studies have reported the antibacterial activity of the EOs from different plants [16,42,44,46]. In contrast, the ethyl acetate fraction was positioned further from 1/MBC at the right lower quadrant, demonstrating the low activity for this fraction. However, several metabolites have been identified in the similar quadrant such as cubebin, yatein, hinokinin, dihydrocubebin, dihydroclusin, cubebinin and magnosalin (lignans). It can be deduced that ethyl acetate fraction has higher content of lignans compared with the rest of extracts and fractions. Extracts of methanol, ethanol, hexane and hexane fraction are positioned close to each other at the left quadrant. Interestingly, cubebol and ledol were also identified in this quadrant along with other metabolites such as *p*-cymene and piperidine. Only 62.9% separation occurred by PC1, and this explained the present of cubebol and ledol on both left and right upper quadrants. The reason that extracts of methanol, ethanol, hexane, and hexane fraction appeared close to each other in biplot might due to their similar bioactivities in MBC towards *S. sobrinus*.

**Figure 4:**
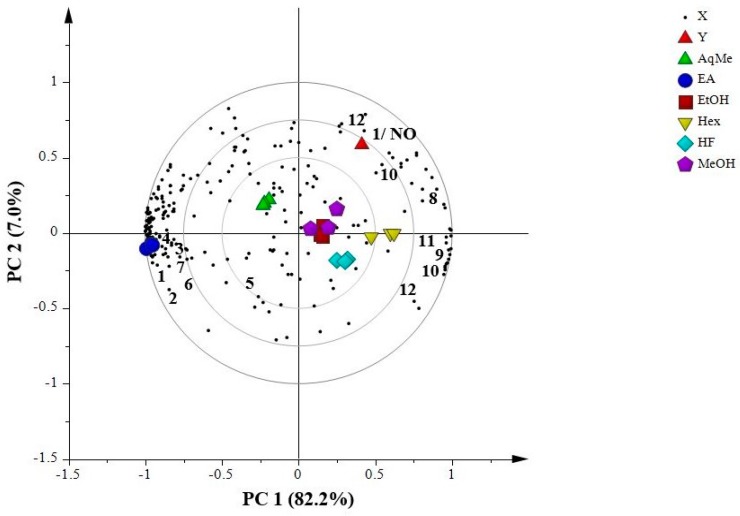
The biplot demonstrates the correlation between extracts and fractions of *P. cubeba* L. at concentration 62.5 µg/mL with anti-inflammatory activity on nitrite oxide (NO) production. MeOH: methanol extract; EtOH: ethanol extract; Hex: hexane extract; HF: hexane fraction; EA: ethyl acetate fraction; AqMe: aqueous methanol fraction. The numbers indicate the identified putative metabolites. **1**: cubebin; **2**: yatein; **3**: hinokinin; **4**: dihydrocubebin; **5**: dihydroclusin; **6**: cubebinin; **7**: magnosalin; **8**: *p*-cymene; **9**: piperidine; **10**: cubebol; **11**: d-germacrene; **12**: ledol. X: metabolites in *P. cubeba* L., Y: 1/NO.

Figure 4 shows the PLS biplot that represents correlation between the NO anti-inflammatory activity assay with the respective NMR data.

As shown in Figure 4, the 1/NO located at the right upper quadrant alongside with methanol extract and ethanol extract, implying the high correlation between the activity and these extracts. The metabolites that appeared to be in the same quadrant are *p*-cymene, cubebol and ledol. *p*-cymene along with other EOs that were previously reported to have anti-inflammatory activity [47,48]. It has been postulated that EOs suppressed the NO production in RAW 264.7 murine macrophage cells by inactivation of NF-κB, the transcription factors responsible for the expression of iNOS in murine macrophages [49]. The in vivo anti-inflammatory activity of EOs in *P. cubeba* L. has been previously reported by [50]. Despite the three identified metabolites in the right upper quadrant, the biplot shows that there are still many metabolites of interest, whose responsibility for the anti-inflammatory activities remains unidentified. Distribution of the groups of samples is an aqueous methanol at the left upper quadrant with no identified metabolites. Meanwhile, hexane extract and hexane fraction were located at the right lower quadrant, wherein piperidine, cubebol, d-germacrene and ledol were identified. Separation by PC2 was only 7.0%, due to the presence of cubebol and ledol on both right upper and lower quadrant. The ethyl acetate fraction followed the same trend as in 1/MBC, whereby the fraction located far from 1/NO with the metabolites from the lignans group identified as cubebin, yatein, hinokinin, dihydrocubebin, dihydroclusin, cubebinin and magnosalin. In summary, only three metabolites in aqueous methanol fraction were identified in this study; cubebol, d-germacrene and ledol that are responsible for the antibacterial activity, MBC against *S. sobrinus.* Meanwhile, cubebol, ledol and *p*-cymene contributed to the anti-inflammatory activity by suppression of NO production and these metabolites were detected higher in methanol and ethanol extracts. The EOs group could be seen as possessing a more significant antibacterial and anti-inflammatory activity compared to the lignans of *P. cubeba* L. extracts and fractions as samples polar solvents have higher antibacterial and anti-inflammatory activity. According to Figure 3 and Figure 4, it can be inferred that ethyl acetate is the best solvent for liquid–liquid partition for extracting lignans from a methanol extract of *P. cubeba* L. in comparison to hexane and aqueous methanol.

Supervised techniques generally tend to over fit, particularly in metabolomics studies, whereby the number of variables is larger, which increases the chance of false correlation. Proper model validation is needed to ensure the model reliability and identification of significant metabolites. In this study, the goodness of fit, prediction of Y and permutation test were used to validate the PLS model [40,51,52]. The significance of model’s fitness is described by the R2 value which demonstrates the quality of Y variables in the model, whereas the predictive quality is explained by the Q2 value. The model has better performance in term of goodness of fit and predictive quality, when the value of R2 and Q2 are near to 1. A permutation test with 100 permutations was done to validate the model. To be a valid model, R2Y and Q2Y intercepts should be less than 0.3–0.4 and 0.05, respectively [53]. PLS model validation for 1/MBC and 1/NO was illustrated in Supplementary Figure S13. The autofit of the PLS model for 1/MBC resulted in two components, exhibited the goodness of fit for Y variables at R2Y = 0.552 and the goodness of prediction of the model with respect to Y variables at Q2 = 0.228. A permutation test on the second component of PLS for 1/MBC showed the R2 and Q2 intercepts fulfill the criteria by having values of 0.212 and −0.182, respectively. Supplementary Figure S13 shows the observed versus predicted plots, with the value for the regression coefficient for 1/MBC at 0.55. 1/MBC model regarded as a rather poor-quality model and only at best to indicate the possible trend. Meanwhile, four components observed in the autofit for the 1/NO PLS model was with value of R2Y = 0.882 and Q2 = 0.658. A permutation test on 1/NO shows Y-axis intercepts at 0.353 and −0.465 for R2 and Q2, respectively. Supplementary Figure S13 showed the observed versus predicted plots, with the value for the regression coefficient for 1/NO at 0.88, which marked that the obtained PLS can be used as the prediction model. 

## 3. Materials and Methods

### 3.1. Materials

Extraction and liquid–liquid partition solvents, methanol, ethanol, hexane and ethyl acetate, were supplied by Systerm, ChemAR, (Kielce, Poland). Media used for antibacterial activity were: Mueller-Hinton broth (MHB) and Mueller-Hinton agar (MHA) were provided by Oxoid Ltd. (Basingstoke, UK). Clorhexidine (CHX) was purchased from Sigma Aldrich Co. (St. Louis, MO, USA). *Streptococcus mutans* KCCM3309 strains were obtained from Korean Culture Center of Microorganism (Daejon, Korea). *Streptocccos sobrinus* ATCC33478 and *Actinomyces viscosus* ATCC15987 were obtained from American Type Culture Collection (Gaithersburg, MD, USA). Media related to cell culture: Dulbecco’s Modified Eagle Medium (DMEM) containing HEPES and l-glutamine with and without phenol red, penicillin streptomycin antibiotic solutions, fetal bovine serum (FBS), 3-(4,5-dimethylthiazol-2-yl)-2,5-diphenyltetrazolium bromide (MTT) and TrypLE Express enzyme were supplied by Gibco (Gaithersburg, MD, USA). Curcumin, interferon-gamma (IFN-γ), lipopolysaccharide (LPS) and phosphate-buffered saline (PBS) were obtained from Sigma Aldrich Co. (St. Louis, MO, USA). The murine macrophage cells line, RAW 264.7 was purchased from the American Type Culture Collection (Gaithersburg, MD, USA). The reagents used for ^1^H-NMR were potassium dihydrogen phosphate (KH_2_PO_4_) provided by Stream Chemicals (Newburyport, MA, USA), sodium-3-trimethylsilylpropionate TMSP-2,2,3,3-*d*_4_ (D,98%), deuterium oxide (D_2_O), sodium deuterium oxide (NaOD) from Cambridge Isotope Laboratories (Tewksbury, MA, USA) and methanol-*d*_4_ (CD_3_OD) obtained from Merck (Darmstadt, Germany).

### 3.2. Plant Materials

Dried berries of *P. cubeba* L. were obtained from a traditional herbal market in Pasar Baru Bandung, Indonesia, in November 2014. Samples were identified by a researcher Associate Prof. Dr. Yaya Rukayadi from Institute of Bioscience (IBS) and the voucher specimen (HBG10PC01) has been deposited in the herbarium of the Laboratory of Natural Products, Institute of Bioscience (IBS), Universiti Putra Malaysia (UPM).

### 3.3. Solvent Extraction of P. cubeba *L*.

An amount of 100 g of dried berries of *P. cubeba* L. (except for methanol in which 850 g was used) was pulverized to a coarse powder. The samples were extracted with 400 mL of each solvent for 48 h at room temperature with occasional shaking. Whatman filter paper No.2 (Whatman International Ltd., Middlesex, UK) was used to filter the plant extracts. A rotary vacuum evaporator (BUCHI Rotavapor R-200, Flawil, Switzerland) was used to concentrate the extracts at 40 °C which yielded three dried *P. cubeba* L. berries crude extracts namely methanol, ethanol and hexane extracts. All the extracts were subjected to freeze drying for 48 h to eliminate water composition in the extracts completely [54]. All plant extracts were stored at 4 °C until further analysis.

### 3.4. Antibacterial Activity Assays

#### 3.4.1. Sample Preparation

Different concentrations of plant crude extracts were prepared using 100% dimethyl sulfoxide (DMSO). For each solvent used, five concentrations were prepared. Initially, 100 mg extract dissolved in 1 mL DMSO yielded a concentration of 100 mg/mL. Dilution was then undertaken to give the concentrations of 50 mg/mL, 25 mg/mL, 12.5 mg/mL, and 6.3 mg/mL. DMSO at 100% concentration was found unable to kill the bacteria tested in this study.

#### 3.4.2. Disc Diffusion Assay 

Briefly, a single uniform colony of freshly prepared inoculum was spread onto MHA plate by using a sterile cotton swab. Sterile paper discs of 6 mm in diameter were punched into inoculated MHA. Each of the paper discs were infused with 10 μL from *P. cubeba* L. extracts/fractions of concentration from 100 mg/mL to 6.3 mg/mL. For positive control, 0.5 mg/mL CHX was used, while DMSO at 100% concentration was used for negative control. The plates were incubated at 37 °C for 24 h. The results were based on the measurement of the diameter of the inhibition zone and recorded in millimeters (mm). The assay was performed in triplicate. All bacterial handling and media preparation were carried out using the aseptic technique in class II biosafety cabinet [55].

#### 3.4.3. Minimum Inhibitory Concentration (MIC) and Minimum Bactericidal Concentration (MBC)

A minimum inhibitory concentration (MIC) was determined by broth microdilution method using sterile 96-wells round bottom microtiter plate with inoculum suspension of all bacteria species, which was adjusted from 10^6^ to 10^8^ CFU/mL. The first column of the wells was filled with 200 μL of MHB, which served as the negative control growth. The second column, positive control growth column was filled with 200 μL of bacterial suspension. The microdilution was performed at extracts/fractions concentration ranged from 50 mg/mL in column twelve to 0.1 mg/mL in column three. Concentrations for the CHX ranged from 250 μg/mL, in column twelve to 0.49 μg/mL in column three. Plate was incubated for 24 h at 37 °C. The MIC value was determined by the lowest concentration of the extract that produced no visible growth on wells.

Minimum bactericidal concentration (MBC) was determined by sub-culturing suspension from each MIC wells onto MH agar plate. A volume of 10 µL from all the wells from column one to 12 was pipetted onto the agar plates. The plates were incubated at 37 °C for 24 h or until growth seen on growth control plates. MBC is defined as the lowest concentration of antibacterial agent at which no growth occurs in the MH agar plates. All bacterial handling and media preparation were carried out using the aseptic technique in class II biosafety cabinet [56].

### 3.5. Liquid–Liquid Partition

The methanol crude extract of *P. cubeba* L. was subjected to liquid–liquid partition by mixing 20 g of the extract with 100 mL methanol and 200 mL water (1:2 ratios) in a 1 L separating funnel. Subsequently, 500 mL hexane was added, and the solution was mixed by gentle swirling in which two layers were formed. The upper layer which was the hexane layer was collected and the aqueous methanol layer was kept in the funnel. Another 500 mL of hexane was added, and the procedure was repeated several times or until the color of the fraction became less intense. The same process was repeated using ethyl acetate. Each fraction obtained, including aqueous methanol fraction was evaporated to dryness and freeze-dried.

### 3.6. Evaluation of Cytotoxic and Anti-Inflammatory Activity Using RAW 264.7 Murine Macrophage Cells Line

#### 3.6.1. Sample Preparation

Six samples which were extracts of methanol, ethanol, hexane, and fractions of hexane, ethyl acetate and aqueous methanol, were freshly prepared prior to the experiment. A 2 mg of each sample was weighed and mixed with 20 µL DMSO. The sample was dissolved using vortex. 1.98 mL of white DMEM was added and dissolved by using 1 mL micropipette to give a concentration of 1000 µg/mL in 1% DMSO. Another five Eppendorf tubes were prepared and pre-filled with 1000 mL white DMEM. Two-fold serial dilution was conducted starting from 1000 µg/mL to 31.3 µg/mL.

#### 3.6.2. 3-(4,5-Dimethylthiazol-2-yl)-2,5-diphenyltetrazolium Bromide (MTT) Assay

RAW 264.7 cells culture was conducted according to the methods as recommended by ATCC [57]. The cells were cultured in DMEM supplemented with 10% FBS and 1% penicillin streptomycin at 37 °C in a humidified incubator with 5% CO_2_. The cell’s media were changed daily and passaged until 80% to 90% confluence condition. PBS were used to wash the cells and they were decanted afterward. Cells were harvested by using TrypLE Express enzyme and fresh medium was added. A cell pellet was obtained by centrifugation at 1000× *g* rpm for 10 min in 4 °C was resuspended in 1 mL of medium. The viable cells were counted using Trypan blue exclusion method. The cells’ concentration was adjusted to 1.0 × 10^5^ cells per well. Each well in the 96-wells plate was seeded with 100 µL cells culture and incubated for 24 h. After 24 h, 100 µL samples (1000 µg/mL to 31.3 µg/mL) were added and the plates were incubated for the next 20 h. MTT solution (20 µL, 5 mg/mL in PBS) was added to the wells. After 4 h incubation, the medium was removed and DMSO was added to dissolve the purple formazan crystals in the cells. The absorbance was measured with a microplate reader at 570 nm. The test sample was considered to be cytotoxic when the percentage of cell viability was less than 80%. Viability of untreated control cells was taken as 100% and the percentage viabilities of treated-cells were calculated by comparison to the control group. The experiments were conducted in triplicate for each concentration. All cell handling and media preparation were carried out using the aseptic technique in class II biosafety cabinet [58].

#### 3.6.3. Nitric Oxide (NO) Production by Extracts and Fractions of *P. cubeba* L. Stimulated with LPS and IFN-γ

All steps in Section 3.6.2 were repeated accordingly until each well in the 96-wells plate was seeded with 100 µL cells culture and incubated for 24 h. After 24 h, the medium was replaced with fresh medium along with the addition of 50 µL of stimuli (10 mg/mL LPS, 10 µg/mL IFN-γ) to the wells. A volume of 100 µL samples (125.0 µg/mL to 1.95 µg/mL) were added and then incubated for next 20 h. NO production was determined by measuring the accumulation of nitrite in the culture supernatant using the Griess reagent. Wells with only curcumin acted as a blank positive control, curcumin with stimuli wells as a positive control, while the only stimuli wells acted as a negative control. Wells containing only medium acted as a blank control. The experiments were conducted in triplicate for each concentration. All cell handling and media preparation were carried out using the aseptic technique in class II biosafety cabinet [59].

### 3.7. Proton Nuclear Magnetic Resonance (^1^H-NMR)

NMR analysis was conducted for all extracts and fractions. Initially, phosphate buffer (90 mM, pH 6.0) was prepared by adding 1.232 g KH_2_PO_4_ with 10 mg of TMSP (0.01%) to 100 mL of deionized water. The solution was dissolved and pH adjusted to 6.0 by mixing 1 mL of NaOD (10 M) with 9 mL of deionized water. The procedures began by transferring 30 mg of sample into a 2 mL Eppendorf tubes containing 0.5 mL CD_3_OD and 0.5 mL phosphate buffer. The solution was then vortexed for 1 min and ultra-sonicated for 20 min without heating at room temperature. The solution was further centrifuged for 5 min at 14,000× *g* rpm. Each sample was prepared in 6 replicates. A volume of 0.6 mL clear supernatant was transferred into 5 mm NMR tubes. A Varian INOVA NMR spectrometer 500 MHz (Varian Inc., Palo Alto, CA, USA) at a running frequency of 499.9 MHz and temperature at 26 °C was used to acquire ^1^H-NMR spectra. The acquisition time of each ^1^H-NMR spectrum was set for 4.29 min, comprising of 64 scans with a spectral width of 20 ppm. The suppression of the large water resonance was accomplished by applying the pre-saturation pulse sequence to the ^1^H-NMR data acquisition. The identification of metabolites was attained by using 2-dimensional (2D) NMR experiments in which include 2D ^1^H *J*-resolved (JRES) and 2D HMBC NMR spectroscopy [60].

### 3.8. Multivariate Data Analysis (MVDA)

Chenomx software (v.8.2) was used to phased and base-line corrected ^1^H-NMR spectra with an equal setting applied for all spectra. The spectra range from δ 0.50 to δ 10.00 were bucketed and automatically binned to ASCII into a region with spectral width δ 0.04 to give a total of 235 integrated regions per NMR spectrum. The residual signals for water and methanol at δ 4.66 to δ 5.00 and δ 3.29 to δ 3.33, respectively, were excluded. After binning the NMR spectra, the average ^1^H-NMR data were subjected to multivariate data analysis, performed using SIMCA (v.13.0, Umetrics AB, Umea, Sweden) with Pareto scaling method. An unsupervised PCA and a supervised PLS method were applied to the ^1^H-NMR data sets to investigate the metabolite multidimensional data sets. The relationship between respective metabolites with anticariogenic activity was assessed with PLS model analysis. A *k*-fold cross-validation method was performed to validate PLS model, with *k* = 7. The Y variable in 1/MBC model represents the MBC (mg/mL) values towards *S. sobrinus,* when treated with *P. cubeba* extracts and fractions. The Y variable the in 1/NO model represents the concentrations of NO produced (μg/mL), when treated with *P. cubeba* extracts and fractions at a concentration of 62.5 μg/mL. The MBC and NO values were presented in 1/MBC and 1/NO due to their parameter having the same trend as the activity in Mediani et al. [60].

### 3.9. Statistical Analysis

For analysis of data, MS Excel (v.2010) and Minitab 17 software (v.17, Minitab Inc., State College, PA, USA) were used. Results are expressed as the mean of 3 replicates ± SD. Analysis of variance (ANOVA) was performed to determine the significant difference which was set at *p* < 0.05.

## 4. Conclusions

Antibacterial activity of *P. cubeba* L. was successfully investigated and the data demonstrated that all tested bacteria were susceptible to *P. cubeba* L. Variation of solvents for extraction, liquid–liquid partition, and the concentrations of extracts and fractions partially affected the antibacterial activity. From the anti-inflammatory assay, the lowest NO production was observed in methanol extract at 62.5 µg/mL with 22.98 µg/mL NO production, suggesting that methanol extract might be a suitable candidate as the anti-inflammatory agent. The result of MTT cell viability assay revealed that the inhibitory effect of methanol extract was not due to cell damage (viability > 80%). Twelve putative metabolites have been identified based on the ^1^H-NMR. PCA confirmed that using different solvents for extraction and liquid–liquid partition yielded different metabolites in *P. cubeba* L. dried berries. The PLS model showed that higher biological activity was related more to the polar solvents, despite some active metabolites being present in the non-polar solvents. The identified metabolites related to the MBC were cubebol, D-germacrene and ledol. Meanwhile, *p*-cymene, cubebol and ledol were observed to contribute to the anti-inflammatory activity in LPS-stimulated RAW 264.7 cells. However, the data obtained from this study is unable to provide the absolute identification of metabolites that is responsible for the antibacterial and anti-inflammatory activities, as there are still many metabolites of interest that remain unidentified. Therefore, future work regarding the identification of these metabolites and their correlation to the antibacterial and anti-inflammatory activities could be proposed. The use of ultra-performance liquid chromatography-tandem mass spectrometry (UPLC-MS/MS), 2D heteronuclear single quantum correlation (HSQC) NMR spectroscopy and 2D total correlation spectroscopy (TOCSY) for identification and confirmation of metabolites are highly recommended.

## Figures and Tables

**Table 1:** Yield of *P. cubeba* L. extracts in different solvents.

Dried Berries (g)	Solvent	Extraction Step	Yield (g)	Yield (%)
100	Methanol	First extraction	18.84	18.84
750	Methanol	Second extraction	164.46	21.93
100	Ethanol	First extraction	15.71	15.71
100	Hexane	First extraction	9.91	9.91

**Table 2:** Results of disc diffusion assay (DDA) for *P. cubeba* L. extracts against oral bacteria.

Inhibition Zone (mm) ± SD									
Conc. (mg/mL)	*Streptococcus mutans* KCCM 3390			*Streptococcus sobrinus* ATCC33478			*Actinomyces viscosus* ATCC15987		
	ME	EE	HE	ME	EE	HE	ME	EE	HE
6.3	9.33 ± 0.58 ^AB a^	10.00 ± 0.00 ^AB a^	8.33 ± 0.29 ^B b^	7.00 ± 0.00 ^C a^	7.00 ± 0.00 ^D a^	7.10 ± 0.17 ^C a^	9.00 ± 0.00 ^C a^	9.00 ± 0.00 ^C a^	9.67 ± 0.58 ^C a^
12.5	9.67 ± 0.58 ^AB a^	9.33 ± 0.58 ^AB a^	10.00 ± 0.00 ^A a^	9.00 ± 0.00 ^B a^	7.00 ± 0.00 ^D c^	8.10 ± 0.10 ^B b^	10.17 ± 0.29 ^BC a^	8.83 ± 0.76 ^C a^	9.67 ± 0.58 ^C a^
25.0	9.00 ± 0.00 ^B b^	9.17 ± 0.29 ^B b^	10.00 ± 0.00 ^A a^	9.00 ± 0.00 ^B a^	8.67 ± 0.58 ^C a^	8.43 ± 0.40 ^B a^	10.67 ± 0.58 ^AB a^	10.00 ± 0.00 ^BC a^	10.50 ± 0.50 ^BC a^
50.0	10.00 ± 0.00 ^A a^	10.00 ± 0.00 ^AB a^	10.00 ± 0.00 ^A a^	11.67 ± 0.58 ^A a^	10.00 ± 0.00 ^B b^	12.03 ± 0.06 ^A a^	11.50 ± 0.50 ^A a^	11.33 ± 0.58 ^A a^	11.33 ± 0.58 ^AB a^
100	10.00 ± 0.00 ^A a^	10.33 ± 0.58 ^A a^*	10.00 ± 0.00 ^A a^	12.17 ± 0.06 ^A a^	11.13 ± 0.15 ^A b^	12.07 ± 0.12 ^A a^	11.67 ± 0.58 ^A ab^	11.17 ± 0.29 ^AB b^	12.67 ± 0.58 ^A a^*
CHX (0.05 mg/mL)	11.00 ± 0.00 *	11.00 ± 0.00 *	11.00 ± 0.00 *	14.00 ± 0.00 *	14.00 ± 0.00 *	14.00 ± 0.00 *	13.33 ± 0.58 *	13.33 ± 0.58 *	13.33 ± 0.58 *

Values are the mean ± SD of replications (*n* = 3). Values with different superscript capital letters within the same columns are significantly different (*p* < 0.05). Values with different superscript small letters within the same rows are significantly different (*p* < 0.05). Means not labelled with asterisk (*) within same column are significantly different from the positive control mean (CHX). ME: methanol extract; EE: ethanol extract; HE: hexane extract.

**Table 3:** Minimum inhibitory concentration (MIC) and minimum bactericidal concentration (MBC) of *P. cubeba* L. extracts.

Bacteria		ME	EE	HE	CHX (µg/mL)
*S. mutans* KCCM3309	MIC (mg/mL) MBC (mg/mL)	0.23 ± 0.15 0.23 ± 0.15	0.10 ± 0.00 0.10 ± 0.00	0.10 ± 0.00 0.10 ± 0.00	1.14 ± 0.75 1.14 ± 0.75
*S. sobrinus* ATCC33478	MIC (mg/mL) MBC (mg/mL)	0.93 ± 0.61 16.67 ± 7.22	0.80 ± 0.69 20.83 ± 7.22	0.87 ± 0.70 20.83 ± 7.22	0.49 ± 0.00 2.77 ± 1.97
*A. viscosus* ATCC15987	MIC (mg/mL) MBC (mg/mL)	0.80 ± 0.69 20.83 ± 7.22	0.47 ± 0.31 20.83 ± 7.22	0.87 ± 0.70 13.54 ± 10.97	1.14 ± 0.75 1.63 ± 0.57

Values are the mean ± SD of replications (*n* = 3). ME: methanol extract; EE: ethanol extract; HE: hexane extract; CHX: chlorhexidine.

**Table 4:** Liquid–liquid partition of *P. cubeba* L. methanol extract.

Methanol Extract (g)	Solvent	Yield (g)	Yield (%)
120	Hexane	46.56	35.82
	Ethyl acetate	13.96	10.74
	Aqueous methanol	2.33	2.59

**Table 5:** Disc diffusion assay of *P. cubeba* L. fractions against oral bacteria.

Inhibition Zone (mm) ± SD									
Conc. (mg/mL)	*Streptococcus mutans* KCCM 3390			*Streptococcus sobrinus* ATCC33478			*Actinomyces viscosus* ATCC15987		
	HF	EAF	AMF	HF	EAF	AMF	HF	EAF	AMF
6.3	7.00 ± 0.00 ^B a^	7.00 ± 0.00 ^B a^	7.00 ± 0.00 ^D a^	7.10 ± 0.17 ^C a^	7.07 ± 0.06 ^D a^	7.00 ± 0.00 ^C a^	7.00 ± 0.00 ^C a^	7.00 ± 0.00 ^C a^	7.00 ± 0.00 ^D a^
12.5	9.33 ± 0.58 ^A a^	9.17 ± 0.29 ^A a^	9.33 ± 0.58 ^C a^	7.13 ± 0.06 ^C a^	7.10 ± 0.10 ^CD a^	7.10 ± 0.10 ^C a^	7.67 ± 0.58 ^C b^	9.33 ± 0.58 ^B a^	9.33 ± 0.58 ^C a^
25.0	9.00 ± 0.00 ^A b^	10.00 ± 0.00 ^A a^	9.83 ± 0.29 ^BC a^	7.33 ± 0.58 ^C b^	7.27 ± 0.06 ^C b^	8.67 ± 0.58 ^B a^	9.33 ± 0.58 ^B b^	10.33 ± 0.58 ^AB ab^	11.00 ± 0.00 ^B a^
50.0	9.00 ± 0.00 ^A b^	9.83 ± 0.29 ^A ab^	10.50 ± 0.50 ^AB a^*	10.00 ± 0.00 ^B a^	10.00 ± 0.00 ^B a^	10.67 ± 0.58 ^A a^	10.00 ± 0.00 ^B b^	10.33 ± 0.58 ^AB ab^	11.17 ± 0.29 ^B a^
100	9.33 ± 0.58 ^A b^	9.66 ± 0.58 ^A b^	11.00 ± 0.00 ^A a^*	11.13 ± 0.12 ^A a^	11.07 ± 0.06 ^A a^	11.20 ± 0.17 ^A a^	12.00 ± 0.00 ^A a^	10.83 ± 0.29 ^A b^	12.33 ± 0.58 ^A a^*
CHX (0.05 mg/mL)	11.00 ± 0.00 *	11.00 ± 0.00 *	11.00 ± 0.00 *	14.00 ± 0.00 *	14.00 ± 0.00 *	14.00 ± 0.00 *	13.33 ± 0.58 *	13.33 ± 0.58 *	13.33 ± 0.58 *

Values are the mean ± SD of replications (*n* = 3). Values with different superscript capital letters within the same columns are significantly different (*p* < 0.05). Values with different superscript small letters within the same rows are significantly different (*p* < 0.05). Means not labelled with asterisk (*) within same column are significantly different from the positive control mean (CHX). HF: hexane fraction; EAF: ethyl acetate fraction; AMF: aqueous methanol fraction.

**Table 6:** Minimum inhibitory concentration (MIC) and minimum bactericidal concentration (MBC) of *P. cubeba* L. fractions.

Bacteria		HF	EAF	AMF	CHX (µg/mL)
*S. mutans* KCCM3309	MIC (mg/mL) MBC (mg/mL)	0.10 ± 0.00 0.10 ± 0.00	0.13 ± 0.06 0.13 ± 0.06	0.10 ± 0.00 0.10 ± 0.00	1.14 ± 0.75 1.14 ± 0.75
*S. sobrinus* ATCC33478	MIC (mg/mL) MBC (mg/mL)	0.67 ± 0.23 25.00 ± 0.00	3.15 ± 2.68 25.00 ± 0.00	1.07 ± 0.46 7.29 ± 4.77	0.49 ± 0.00 2.77 ± 1.97
*A. viscosus* ATCC15987	MIC (mg/mL) MBC (mg/mL)	1.58 ± 1.35 16.67 ± 7.22	1.20 ± 0.69 25.00 ± 0.00	1.07 ± 0.46 12.50 ± 10.83	1.14 ± 0.75 1.63 ± 0.57

Values are the mean ± SD of replications (*n* = 3). HF: hexane fraction; EAF: ethyl acetate fraction; AMF: aqueous methanol fraction.

## Supplementary Materials

The Supplementary Materials available online. And References [61,62,63,64,65,66,67,68,69,70,71] are cited in Supplementary Materials.
