# Supplementary material for: Solvent Extraction and Identification of Active Anticariogenic Metabolites in Piper cubeba L. through 1H-NMR-Based Metabolomics Approach
Source: Molecules. 2018 Jul 16;23(7):1730. doi: 10.3390/molecules23071730 (PMC6099612; doi:10.3390/molecules23071730)
Supplement: Supplementary file 1 [file molecules-23-01730-s001.pdf]

SUPPLEMENTARY MATERIALS

Table S1. ^{1}H NMR characteristic signals of identified putative metabolites in *P. cubeba* L. extracts and fractions.

Metabolites	Reported ^{1}H NMR signals	ME	EE	HE	HF	EAF	AMF
**1. Cubebin**	6.80–6.40 (m)	+	+	+	+	+	+
	5.95 (s)	+	+	+	+	+	+
	4.05 (dd)	+	+	+	+	+	+
	3.72 (dd)	3.74 (m)	3.74 (m)	3.74 (m)	3.73 (m)	3.74 (m)	3.74 (m)
	2.90-2.50 (m)	+	+	+	+	+	+
	2.30-1.90 (m)	+	+	+	+	+	+
**2. Yatein**	6.69 (d, *J*= 8.3)	+ (*)	nd	+ (*)	+ (*)	+ (*)	+ (*)
	6.36 (s)	+	+	+	+	+	+
	5.94 (d, *J*= 1.5)	5.94 (m)	5.94 (m)	5.94 (m)	5.94 (m)	5.94 (m)	5.94 (m)
	5.93 (d, *J*= 1.5)	5.93 (m)	5.93 (m)	5.93 (m)	5.93 (m)	5.93 (m)	5.93 (m)
	3.82 (s)	+	+	+	+	+	+
	2.92-2.89 (m)	nd	nd	nd	nd	+	nd
	2.63-2.48 (m)	+	+	+	+	+	+
**3. Hinokinin**	6.80–6.40 (m)	+	+	+	+	+	+
	5.90 (s)	+	+	+	+	+	+
	2.60 (d, *J*= 7.1)	+	+	+	2.60 (m)	+	+
	2.55 (m)	+	+	+	+	+	+
	2.45 (d, *J*= 8.6)	2.45 (m)	2.45 (m)	2.45 (m)	2.45 (m)	2.45 (m)	2.45 (m)
**4. Dihydro-cubebin**	6.60 (m)	+	+	+	+	+	+
	3.70 (dd, *J*= 1.3, 11.3)	3.74 (m)	3.74 (m)	3.74 (m)	3.73 (m)	3.74 (m)	3.73 (m)
	3.20 (s)	nd	nd	nd	+	+	+
	2.70 (d, *J*= 8.7)	2.70 (m)	2.70 (m)	nd	2.72 (m)	2.72 (m)	2.70 (m)
	2.65 (d, *J*= 8.7)	+	+ (*)	nd	+ (*)	nd	+
	2.56 (d, *J*= 5.7)	2.56 (m)	nd	nd	+ (*)	2.56 (m)	nd
	2.51 (d, *J*= 5.7)	2.50 (m)	+ (*)	nd	+ (*)	2.50 (m)	2.50 (m)
**5. Dihydro-clusin**	6.72 (d, *J*= 8.0)	+ (*)	+ (*)	+ (*)	+ (*)	+ (*)	+ (*)
	6.59 (d, *J*= 8.0)	+	+	+	+	+ (*)	+
	6.36 (s)	+	+	+	+	+	+
	5.92 (s)	+	+	+	+	+	+
	3.84 (s)	+	+	+	+	+	+
	3.80-3.42 (m)	+	+	+	+	+	+
	1.88 (m)	+	+	+	+	nd	nd
**6. Cubebinin**	6.47 (s)	+	+	nd	nd	+	+
	6.40 (s)	+	+	+	+	+	+
	6.36 (s)	+	+	+	+	+	+
	5.27 (s)	+	+	+	+	nd	+
	4.03 (t, *J*= 8.0)	+	+	+	+	+	+
	3.84 (s)	+	+	+	+	+	+
	3.82 (s)	+	+	+	+	+	+
	3.80 (s)	+	+	+	+	+	+
	3.62 (t, *J*= 8.0)	+	+	+	+	+	+
	2.04-2.85 (m)	+	+	+	+	+	+
**7. Magnosalin**	6.46 (s)	+	+	nd	nd	+	+
	3.86 (s)	+	+	nd	nd	+	+
	3.84 (s)	+	+	+	+	+	+
	3.68 (s)	nd	nd	nd	nd	+	+
	1.75 (s)	+	nd	nd	nd	nd	+
	1.19 (d, *J*= 5.5)	+	+	+	+	nd	+
**8. *p*-cymene**	2.87 (m)	+	+	+	+	nd	+
	2.23 (s)	+	+	+	+	+	+

	1.23 (d, *J*= 8.5)	+	+	+	+	+	+
**9. Piperidine**	2.79 (t)	2.76 (m)	2.75 (m)	2.75 (m)	2.75 (m)	2.75 (m)	2.75 (m)
	2.18 (s)	nd	nd	nd	nd	2.19 (s)	nd
	1.58 (t)	1.58 (m)	1.58 (m)	1.58 (m)	1.58 (m)	nd	1.58 (m)
	1.55 (d)	1.56 (m)	1.55 (m)	1.56 (m)	1.56 (m)	nd	1.56 (m)
	1.51 (m)	+	+	+	+	nd	+
**10. Cubebol**	1.84-1.37 (m)	+	+	+	+	nd	+
	1.28 (s)	+	+	+	+	+	+
	0.97 (d, *J*= 6.8)	+ (*)	+ (*)	+ (*)	+ (*)	0.97 (m)	+
	0.94 (d, *J*= 6.5)	+ (*)	+ (*)	+ (*)	+ (*)	0.94 (m)	+
	0.91 (d, *J*= 4.3)	+ (*)	+ (*)	+ (*)	+ (*)	0.91 (m)	+
	0.87 (d, *J*= 3.4)	0.88 (m)	0.88 (m)	0.88 (m)	0.88 (m)	0.87 (m)	0.88 (m)
	0.83 (dd, *J*= 3.4, 3.4)	0.88 (m)	0.88 (m)	0.83 (m)	0.88 (m)	0.87 (m)	0.88 (m)
**11. D-Germacrene**	4.93 (brs)	+	+	+	+	+	+
	2.30- 190 (m)	+	+	+	+	+	+
	1.45 (brs)	+	+	+	+	+	+
	1.30 (m)	+	+	+	+	+	+
	0.88 (d, *J*= 6.7)	0.88 (m)	0.88 (m)	0.88 (m)	0.88 (m)	0.87 (m)	0.88 (m)
	0.84 (d, *J*= 6.8)	+	+	+	+	0.85 (m)	+ (*)
**12. Ledol**	1.65-156 (m)	+	+	+	+	+	+
	1.39 (m)	+	+	+	+	nd	+
	1.15 (s)	+	+	+	+	+	+
	1.02 (s)	+	+	+	+	+	+
	0.98 (s)	+	+	+	+	+	+
	0.92 (d, *J*=7.00)	+ (*)	+ (*)	+ (*)	+ (*)	0.91 (m)	+ (*)

(*) Indicates the identification has been confirm with 2D ^{1}H *J*-resolved (JRES) NMR spectroscopy. (nd): not detected. ME: methanol extract; EE: ethanol extract; HE: hexane extract; HF: hexane fraction; EAF: ethyl acetate fraction; AMF: aqueous methanol fraction. Metabolites reported from; **1:** Souza et al. (2004), **2:** Miyata et al. (1998), **3:** Souza et al. (2005), **4:** Laurentiz et al. (2015), **5** and **6:** Prabhu and Mulchandani, (1985), **7:** Ryu et al. (2002), **8:** Human Metabolome Database (HMDB) 05805, **9:** HMDB 0034301, **10:** Chen et al. (2001), **11:** Mori et al. (1990), **12:** Miyazawa et al. (1994).

Table S2. Nitric oxide (NO) production by extracts and fractions of *P. cubeba* L. stimulated with LPS and IFN-γ in RAW 264.7 cells.

	NO production (μg/mL)					
**Conc.**	**ME**	**EE**	**HE**	**HF**	**EAF**	**AMF**
LPS, IFN-γ	45.53 ± 3.98	45.53 ± 3.98	45.53 ± 3.98	45.53 ± 3.98	45.53 ± 3.98	45.53 ± 3.98
1.95	41.14 ± 3.46	40.49 ± 1.02	n.d	44.32 ± 1.57	44.48 ± 2.70	n.d
3.90	39.33 ± 4.14	35.82 ± 4.52	35.20 ± 9.52	43.13 ± 0.85	42.37 ± 1.69	43.49 ± 2.26
7.81	38.29 ± 3.47	33.13 ± 7.61	34.20 ± 8.94	40.90 ± 0.93	39.28 ± 2.39	40.43 ± 5.85
15.63	31.73 ± 2.95	31.88 ± 5.19	32.99 ± 9.81	37.96 ± 0.48	39.03 ± 1.44	37.79 ± 3.24
31.25	31.16 ± 2.96	24.29 ± 7.36	37.00 ± 5.75	33.72 ± 1.29	36.42 ± 2.09	33.27 ± 2.22
62.50	22.98 ± 5.18	23.13 ± 1.81	28.96 ± 0.54	34.44 ± 0.64	37.68 ± 2.98	27.31 ± 1.83
125.00	n.d	n.d	22.12 ± 6.59	n.d	n.d	18.88 ± 2.15
Control	4.09 ± 0.15	4.09 ± 0.15	4.09 ± 0.15	4.09 ± 0.15	4.09 ± 0.15	4.09 ± 0.15
Curcumin	4.79 ± 0.12	4.79 ± 0.12	4.79 ± 0.12	4.79 ± 0.12	4.79 ± 0.12	4.79 ± 0.12

Values are the mean ± S.D. of replications (n =3). n.d: no data. ME: methanol extract; EE: ethanol extract; HE: hexane extract; HF: hexane fraction; EAF: ethyl acetate fraction; AMF: aqueous methanol fraction.

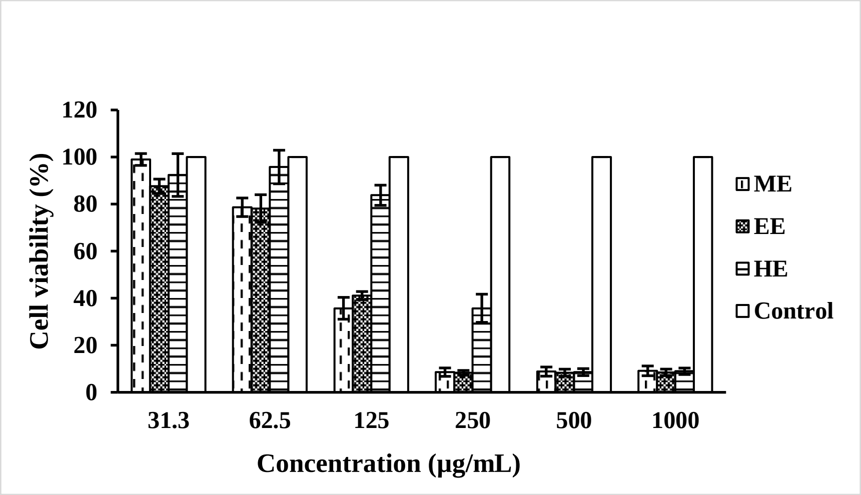

Figure S1. Percentage of cell viability (%) treated with different *P. cubeba* L. extracts. Values are the mean ± S.D. of replications (n=3). ME: methanol extract; EE: ethanol extract; HE: hexane extract.

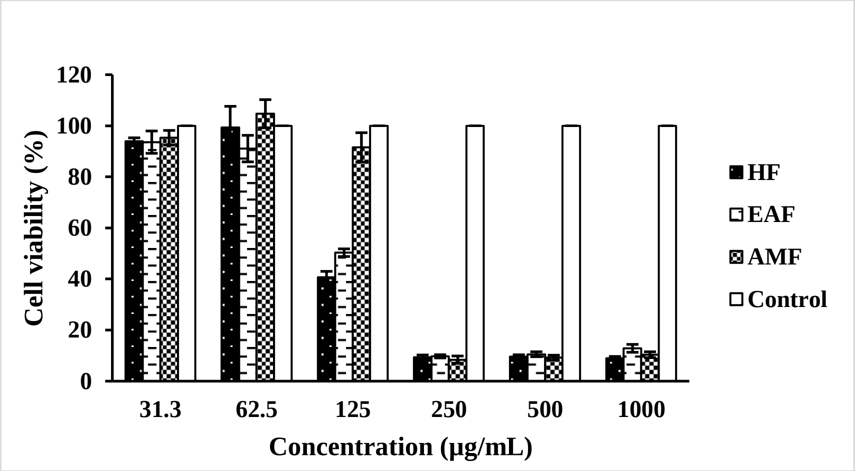

Figure S2. Percentage of cell viability (%) treated with different *P. cubeba* L. fractions. Values are the mean ± S.D. of replications (n=3). HF: hexane fraction; EAF: ethyl acetate fraction; AMF: aqueous methanol fraction.

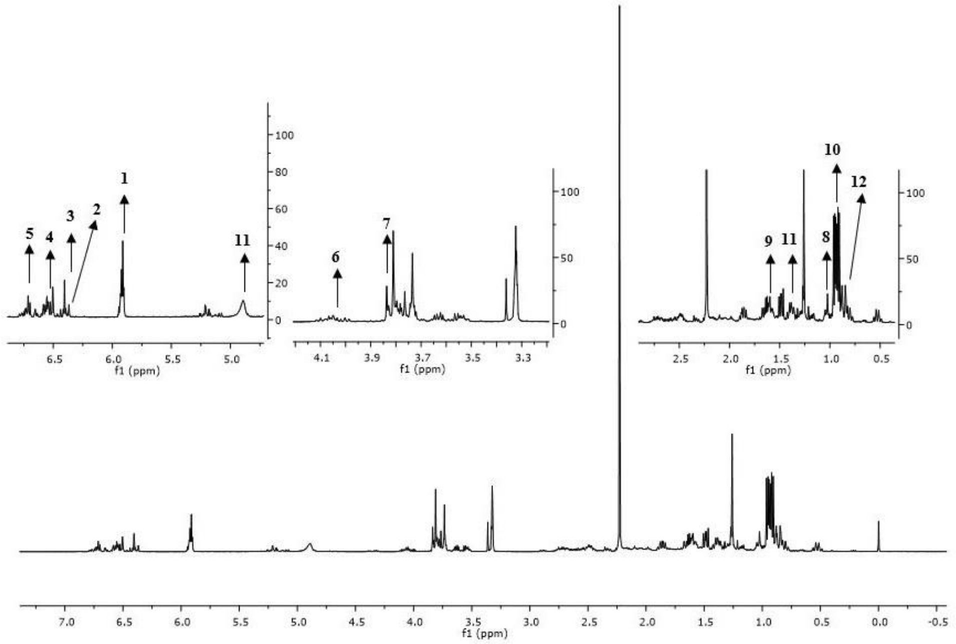

Figure S3. The ^{1}H NMR spectra of ethanol extract of *P. cubeba* L.. The numbers indicate the identified putative metabolites. **1**: cubebin; **2**: yatein; **3**: hinokinin; **4**: dihydrocubebin; **5**: dihydroclusin; **6**: cubebinin; **7**: magnosalin; **8**: *p*-cymene; **9**: piperidine; **10**: cubebol; **11**: D-germacrene; **12**: ledol.

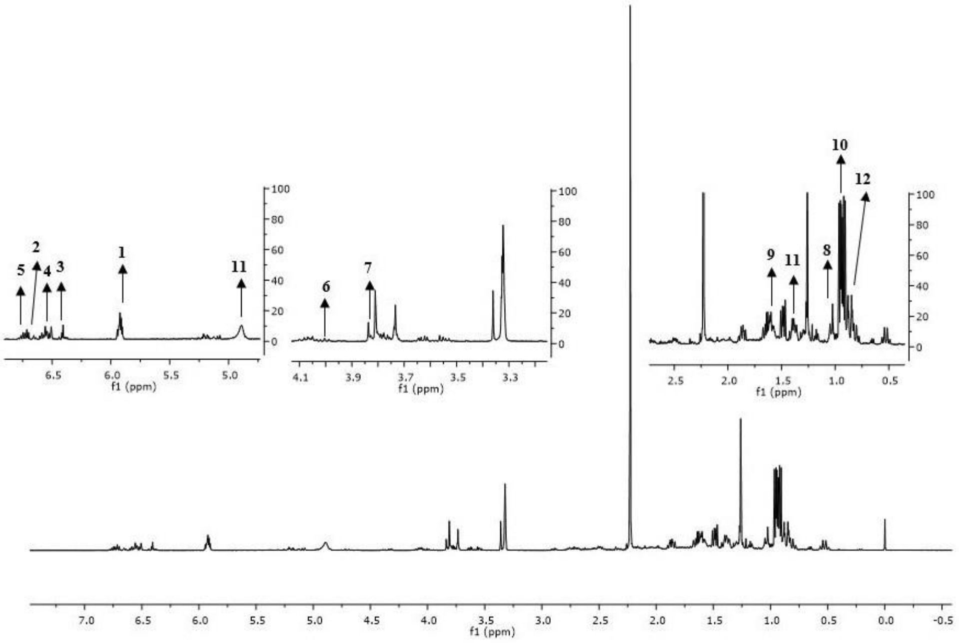

Figure S4. The ^{1}H NMR spectra of hexane extract of *P. cubeba* L.. The numbers indicate the identified putative metabolites. **1**: cubebin; **2**: yatein; **3**: hinokinin; **4**: dihydrocubebin; **5**: dihydroclusin; **6**: cubebinin; **7**: magnosalin; **8**: *p*-cymene; **9**: piperidine; **10**: cubebol; **11**: D-germacrene; **12**: ledol.

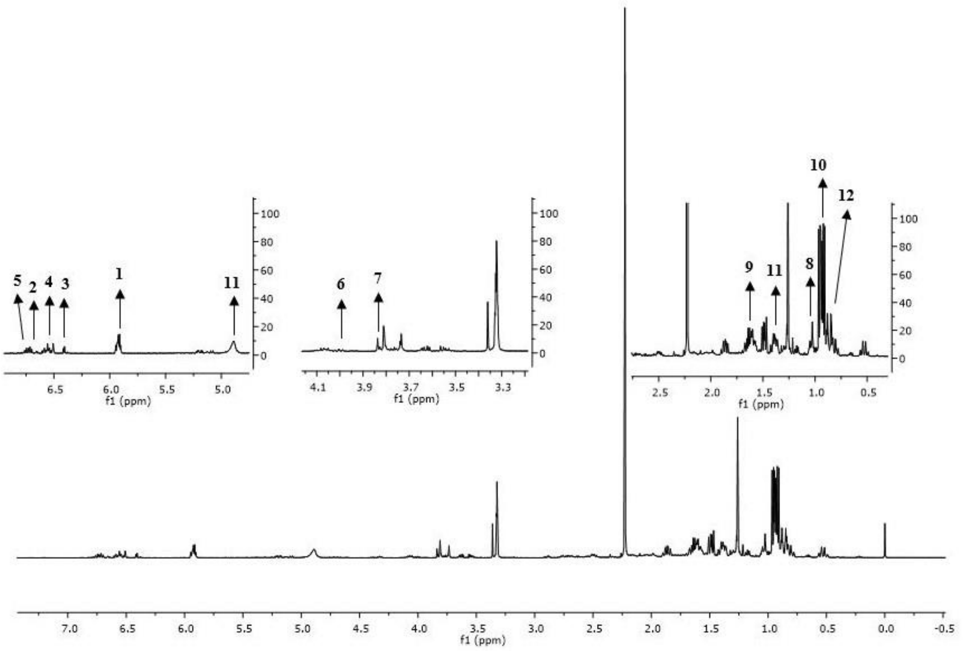

Figure S5. The ^{1}H NMR spectra of hexane fraction of *P. cubeba* L.. The numbers indicate the identified putative metabolites. **1**: cubebin; **2**: yatein; **3**: hinokinin; **4**: dihydrocubebin; **5**: dihydroclusin; **6**: cubebinin; **7**: magnosalin; **8**: *p*-cymene; **9**: piperidine; **10**: cubebol; **11**: D-germacrene; **12**: ledol.

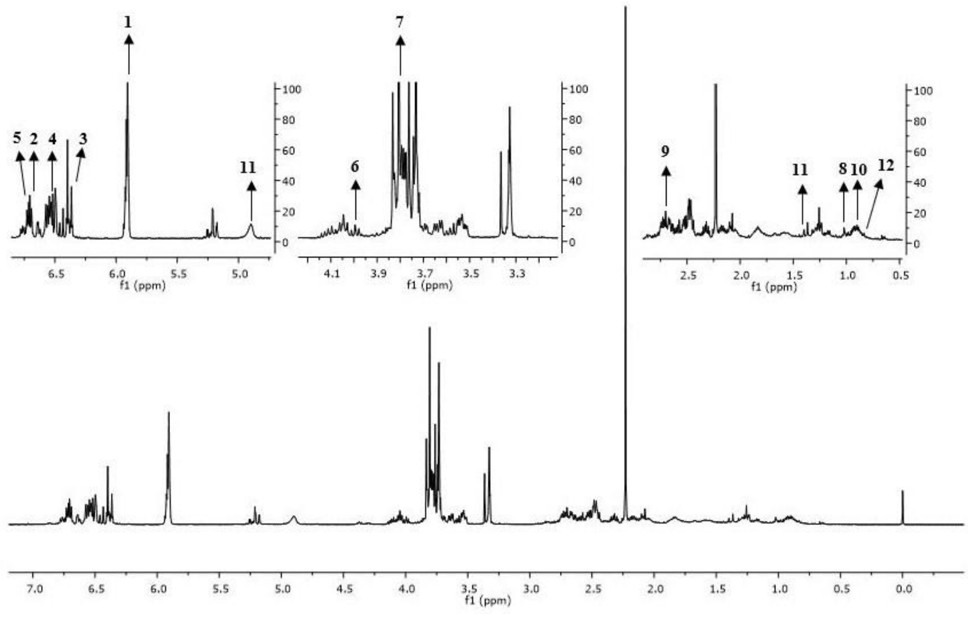

Figure S6. The ^{1}H NMR spectra of ethyl acetate fraction of *P. cubeba* L.. The numbers indicate the identified putative metabolites. **1**: cubebin; **2**: yatein; **3**: hinokinin; **4**: dihydrocubebin; **5**: dihydroclusin; **6**: cubebinin; **7**: magnosalin; **8**: *p*-cymene; **9**: piperidine; **10**: cubebol; **11**: D-germacrene; **12**: ledol.

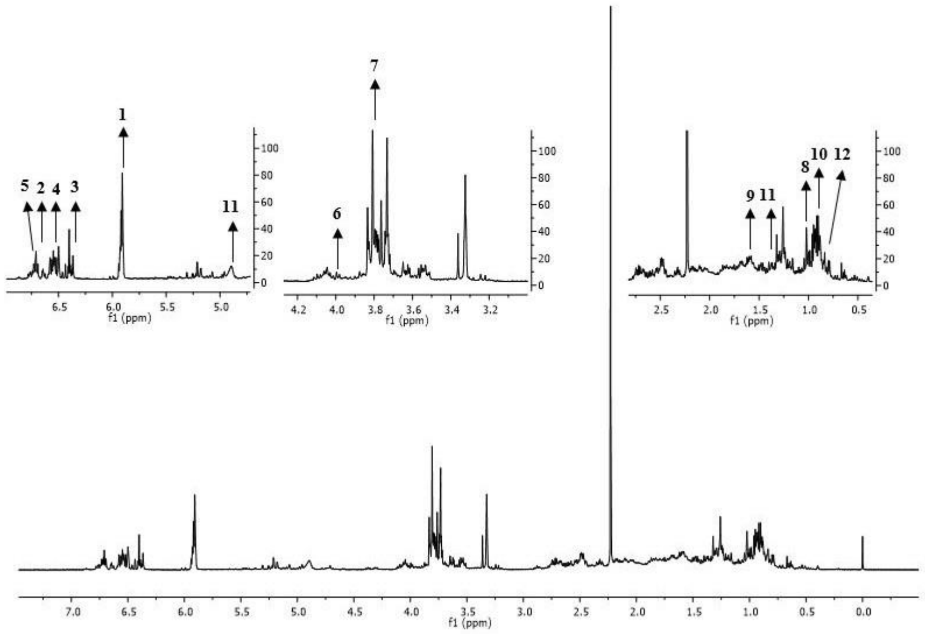

Figure S7. The ^{1}H NMR spectra of aqueous methanol fraction of *P. cubeba* L.. The numbers indicate the identified putative metabolites. **1**: cubebin; **2**: yatein; **3**: hinokinin; **4**: dihydrocubebin; **5**: dihydroclusin; **6**: cubebinin; **7**: magnosalin; **8**: *p*-cymene; **9**: piperidine; **10**: cubebol; **11**: D-germacrene; **12**: ledol.

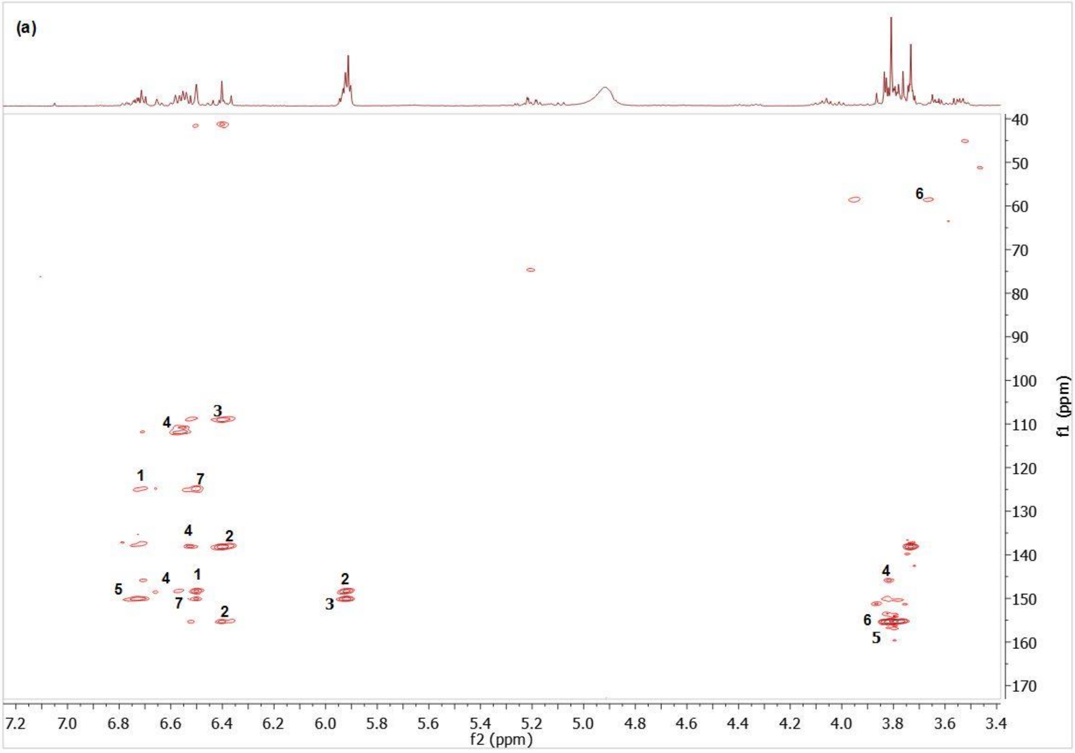

Figure S8 (a). Representative heteronuclear bond correlation (HMBC) spectrum of methanol extract of *P. cubeba* L. in the range of δ 3.40-δ 7.00 of ^{1}H and δ 50-δ 170 of [13]C. The numbers indicate the identified putative metabolites. **1**: correlation of H-7′/C-6′, H-2′/C-3′ of cubebin; **2**: correlation of H-2/C-4, H-6/C-5/, H-10′/C-3′ of yatein; **3**: correlation of H-10/C-4, H-6/C-2 of hinokinin; **4**: correlation of H-2′/C-3′, H-2′/C-1′, H-2′/C-5, H-4/C-3 of dihydrocubebin; **5**: correlation of H-6′/C-4′, Ar-OCH_3/C-3′′ of dihydroclusin; **6**: correlation of H-5/C-3, Ar-OCH_3/C-3′ of cubebinin; **7**: correlation of H-3′/C-1′, H-3′/C-4′ of magnosalin.

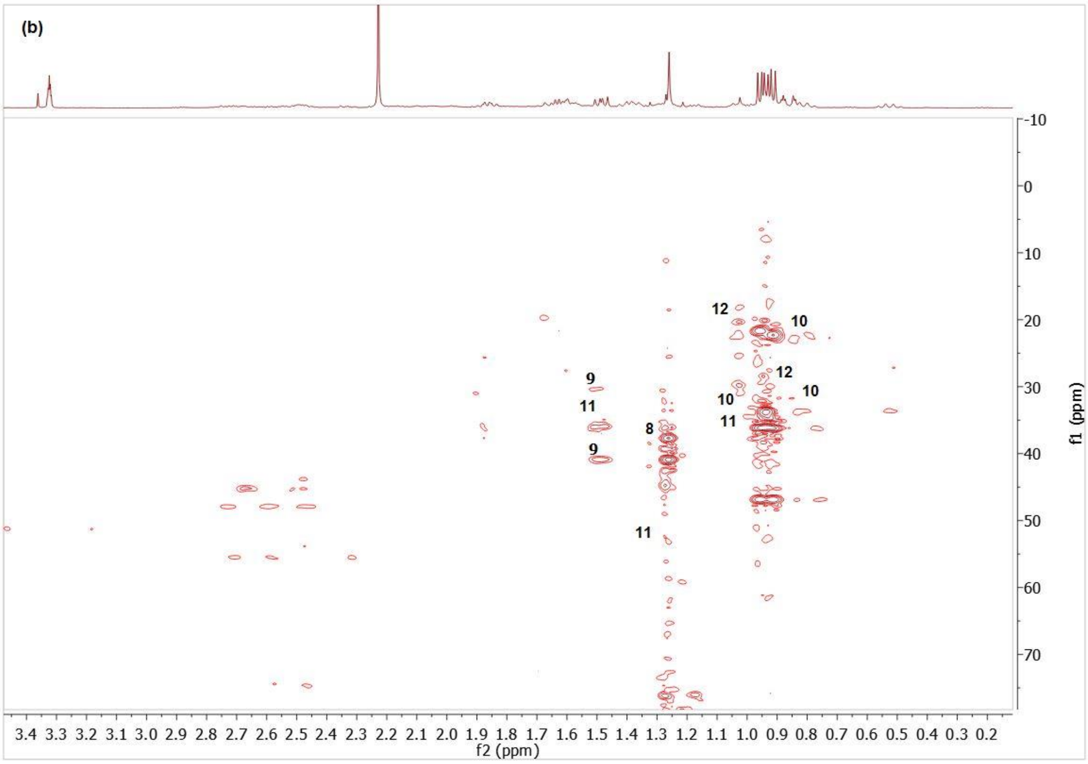

Figure S8 (b). Representative heteronuclear bond correlation (HMBC) spectrum of methanol extract of *P. cubeba* L. in the range of δ 0.20-δ 2.00 of ^{1}H and δ 10-δ 70 of ^{13}C. The numbers indicate the identified putative metabolites. **8**: correlation of H-9/C-7, H-9/C-4 of *p*-cymene; **9**: correlation of H-4/C-5, H-4/C-2 of piperidine; **10**: correlation of H-14/C-10, H-14/C-1, H-5/C-1, H-5/C-6 of cubebol; **11**: correlation of H-13/C-11, H-8/C-7, H14-C9 of D-germacrene; **12**: correlation of H-12/C-11, H-13/C-7 of ledol.

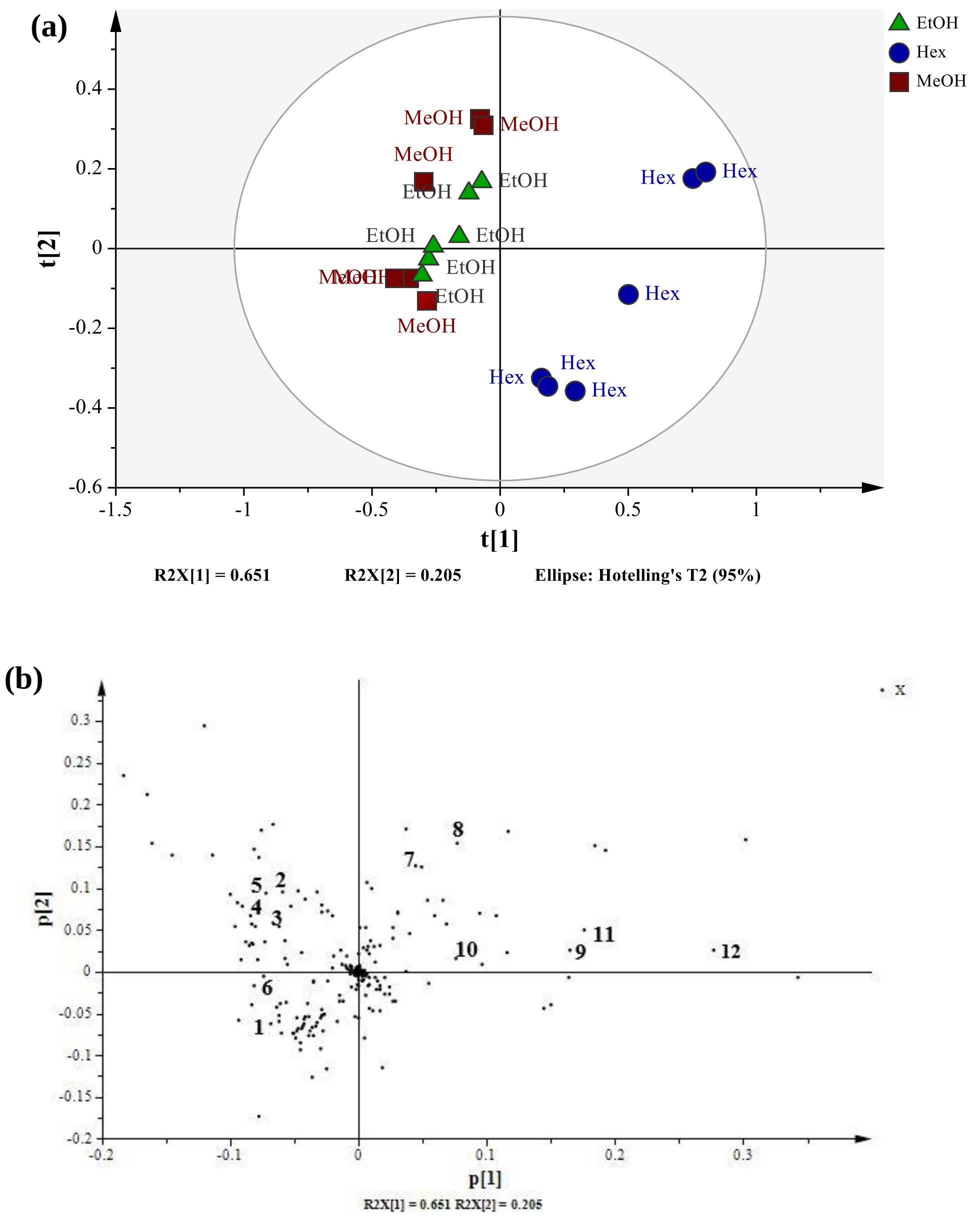

Figure S9. Principal component analysis (PCA) (**a**) score plot (PC1 vs. PC2) and (**b**) loading scatter plot of ^{1}H NMR data of different extracts of *P. cubeba* L. The numbers indicate the identified putative metabolites. **1**: cubebin; **2**: yatein; **3**: hinokinin; **4**: dihydrocubebin; **5**: dihydroclusin; **6**: cubebinin; **7**: magnosalin; **8**: *p*-cymene; **9**: piperidine; **10**: cubebol; **11**: D-germacrene; **12**: ledol, X: metabolites in *P. cubeba* L.

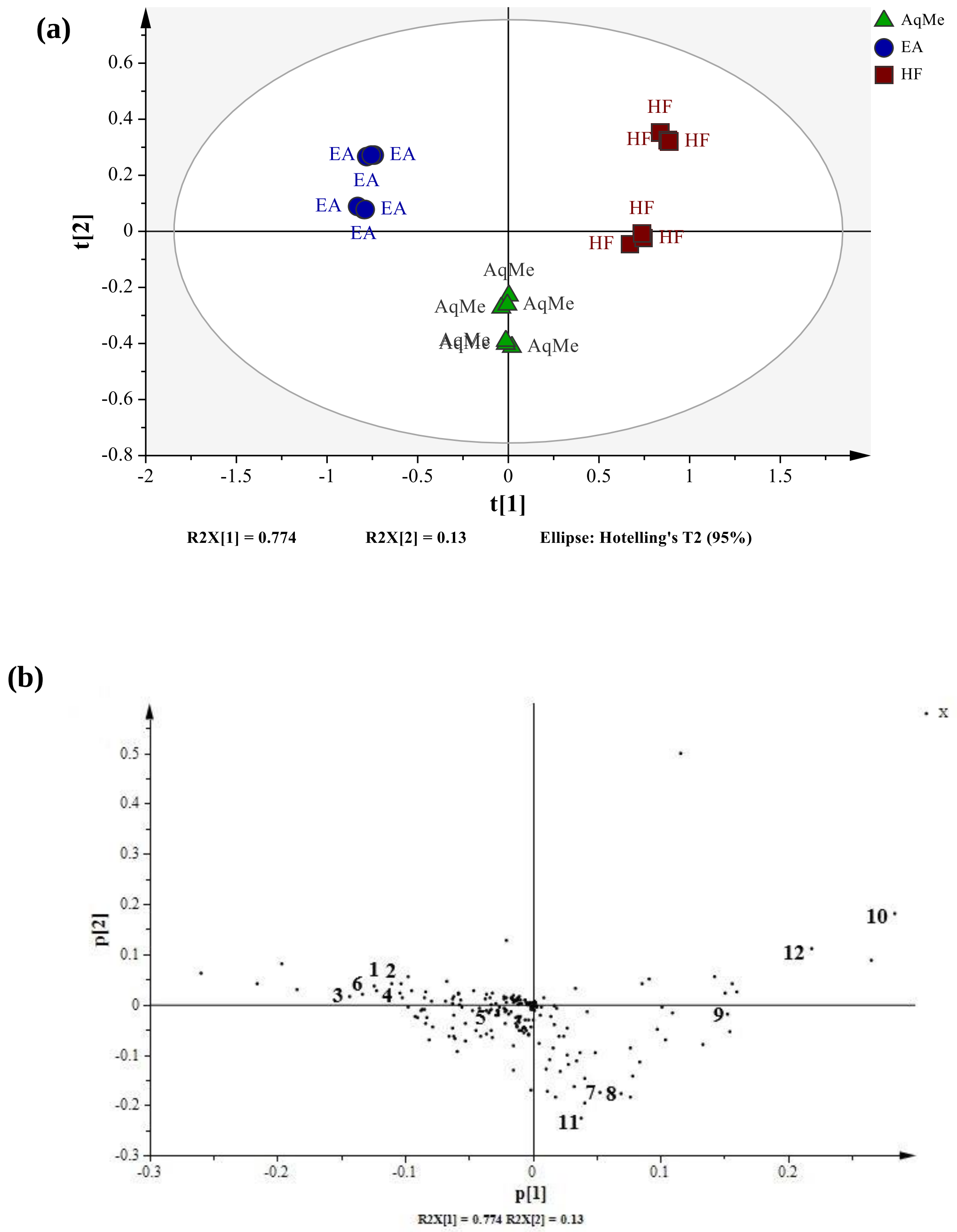

Figure S10. PCA (**a**) score plot (PC1 vs. PC2) and (**b**) loading scatter plot of ^{1}H NMR data of different fractions of *P. cubeba* L. The numbers indicate the identified putative metabolites. **1:** cubebin; **2:** yatein; **3:** hinokinin; **4:** dihydrocubebin; **5:** dihydroclusin; **6:** cubebinin; **7:** magnosalin; **8**: *p*-cymene; **9:** piperidine; **10:** cubebol; **11:** D-germacrene; **12:** ledol, X: metabolites in *P. cubeba* L.

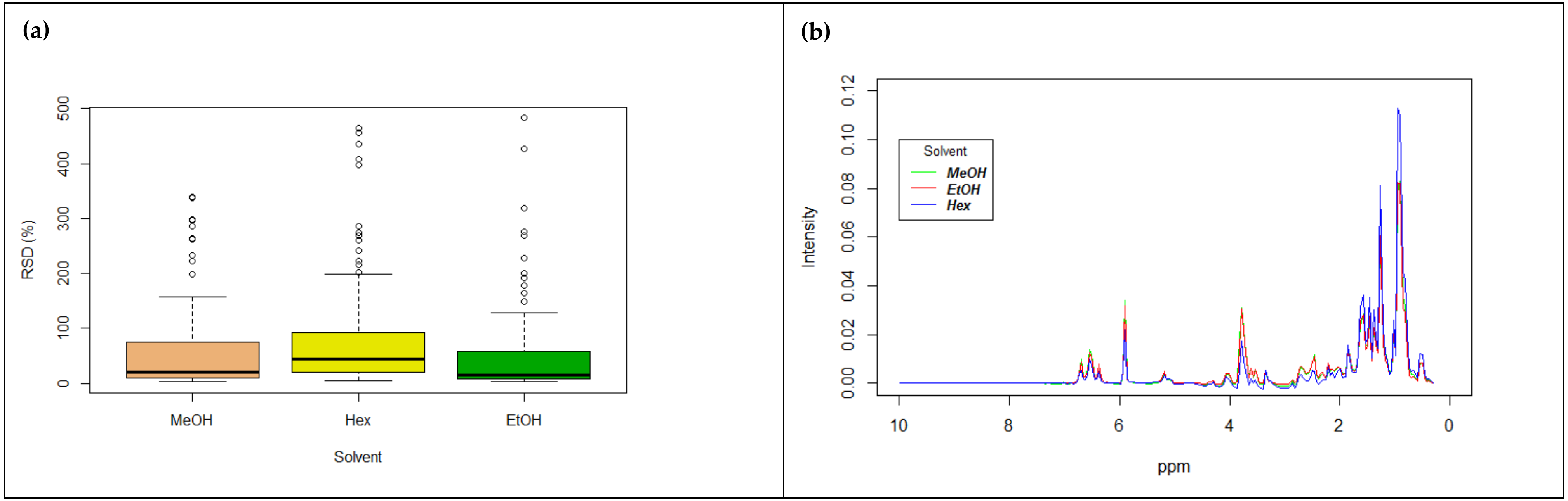

Figure S11. Calculation of spectral relative standard deviation (RSD) values of different solvents of *P. cubeba* L. extracts. **(a)** summary of RSD values **(b)** Three overlaid ^{1}H NMR spectra of *P. cubeba* L. using different extraction solvents (bin width of 0.04 ppm).

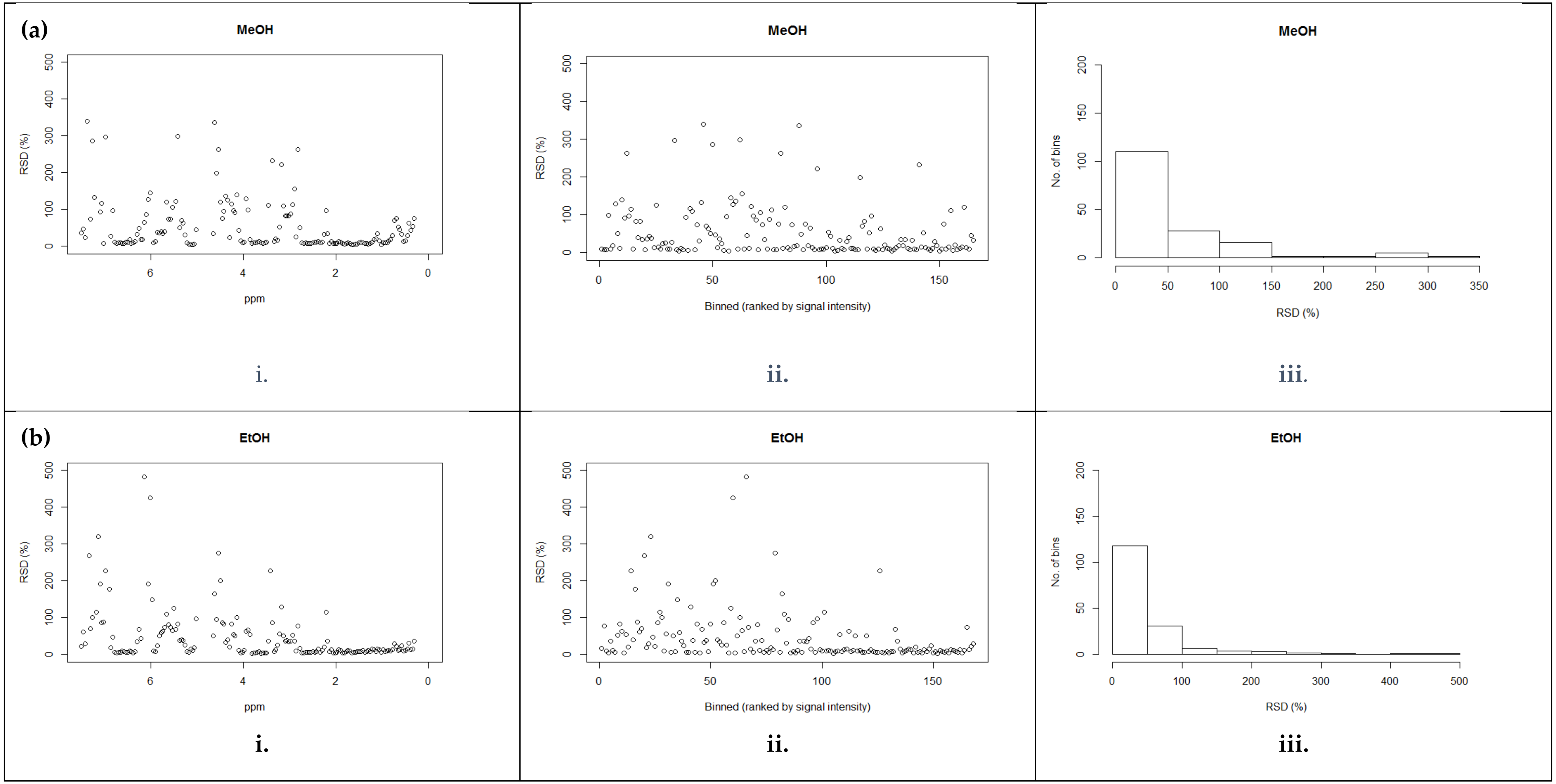
(a)
MeOH
RSD (%)
ppm
i.
MeOH
RSD (%)
Binned (ranked by signal intensity)
ii.
MeOH
No. of bins
RSD (%)
iii.
(b)
EtOH
RSD (%)
ppm
i.
EtOH
RSD (%)
Binned (ranked by signal intensity)
ii.
EtOH
No. of bins
RSD (%)
iii.

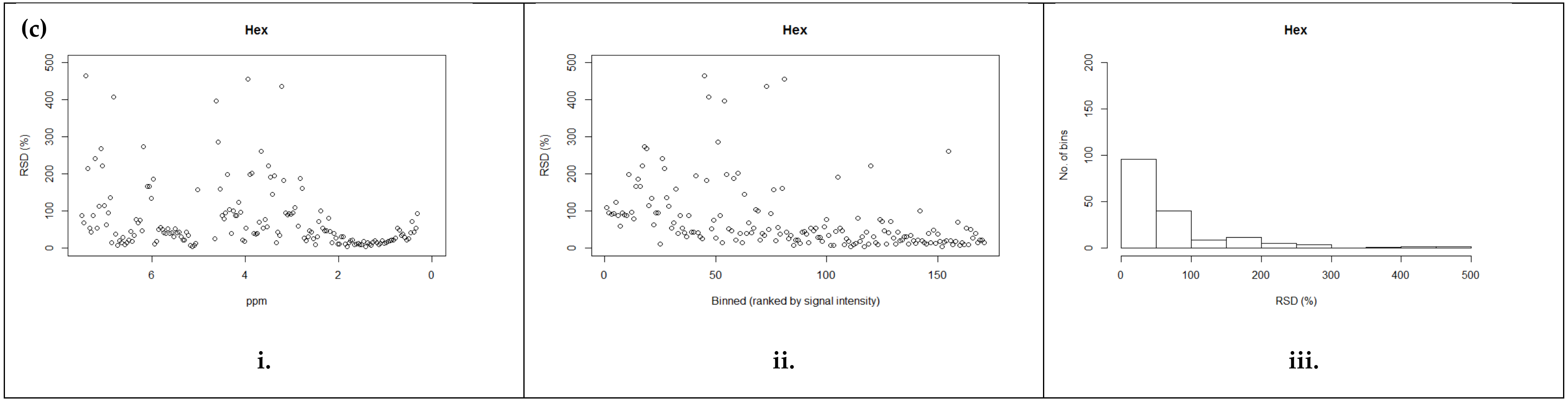

Figure S12. RSD calculation for each extract. The RSD results showed the values range from 46.82-75.88% for all extracts. **(a)** Methanol extract, **(b)** Ethanol extract, **(c)** Hexane extract; i. RSD values, ii. RSD values (ranked according to bin signal intensity), iii. Histogram of RSD values showing a right-skewed distribution, Mean RSD (%) and standard deviation for different extraction solvents, MeOH: 53.20 ± 69.16, EtOH: 46.82 ± 72.51, Hex: 75.88 ± 88.31.

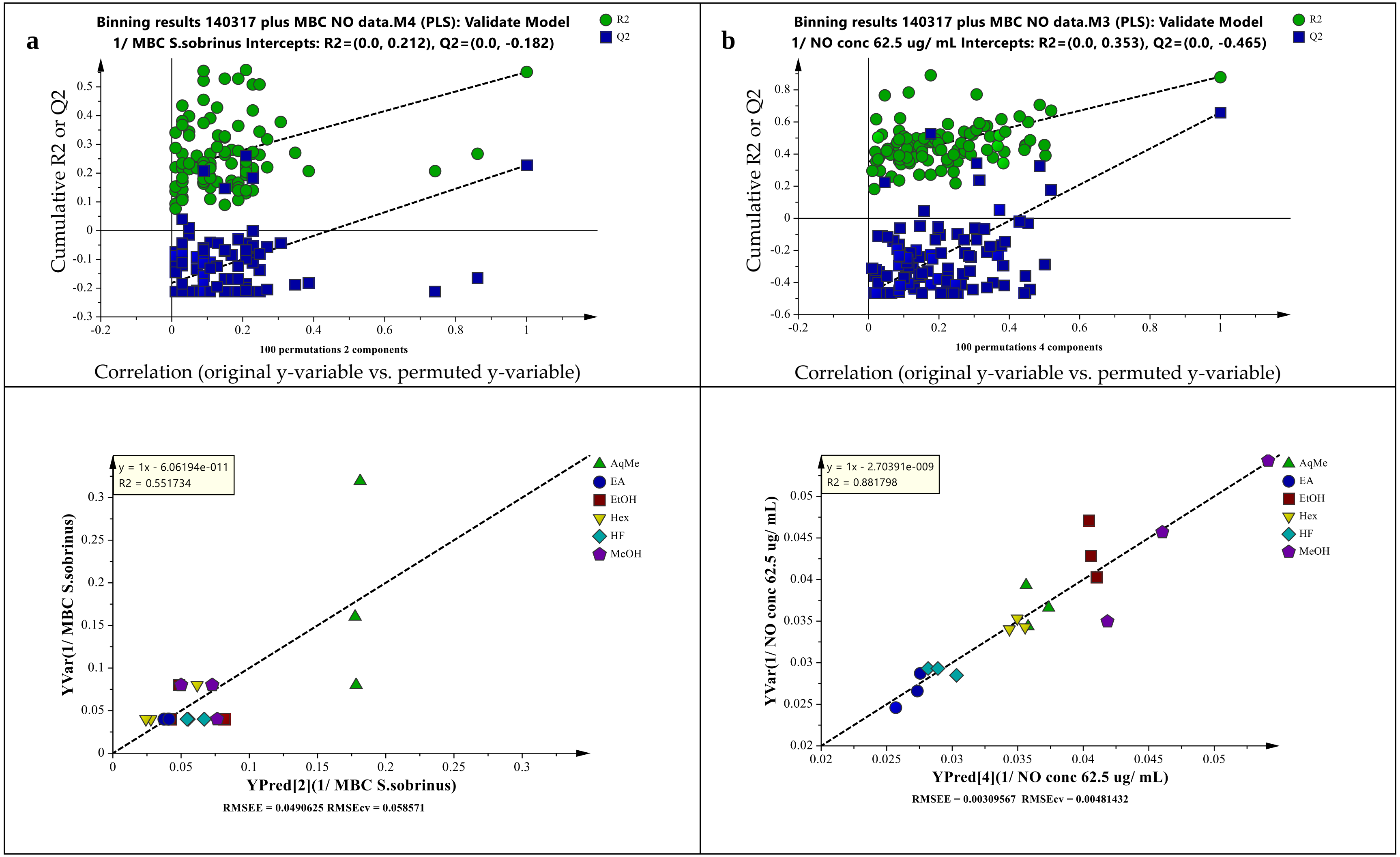

Figure S13. Validation of partial least squares (PLS) model using permutation test and observed and predicted plots for (a) minimum bactericidal concentration (1/MBC), (b) nitrite oxide (1/NO) production.
